# Flexible, Free-Standing Polymer Membranes Sensitized by CsPbX3 Nanocrystals as Gain Media for Low Threshold, Multicolor Light Amplification

**DOI:** 10.1021/acsphotonics.2c00426

**Published:** 2022-06-24

**Authors:** Modestos Athanasiou, Andreas Manoli, Paris Papagiorgis, Kyriacos Georgiou, Yuliia Berezovska, Andreas Othonos, Maryna I. Bodnarchuk, Maksym V. Kovalenko, Grigorios Itskos

**Affiliations:** † Experimental Condensed Matter Physics Laboratory, Department of Physics, University of Cyprus, Nicosia 1678, Cyprus; ‡ Institute of Inorganic Chemistry, Department of Chemistry and Applied Biosciences, ETH Zürich, Zürich CH-8093, Switzerland; § Laboratory for Thin Films and Photovoltaics, Empa—Swiss Federal Laboratories for Materials Science and Technology, Überlandstrasse 129, Dübendorf CH-8600, Switzerland; ∥ Department of Physics, Laboratory of Ultrafast Science, University of Cyprus, Nicosia 1678, Cyprus

**Keywords:** lead halide perovskites, nanocrystals, amplified spontaneous emission, solution-processed lasers, polymer resonators

## Abstract

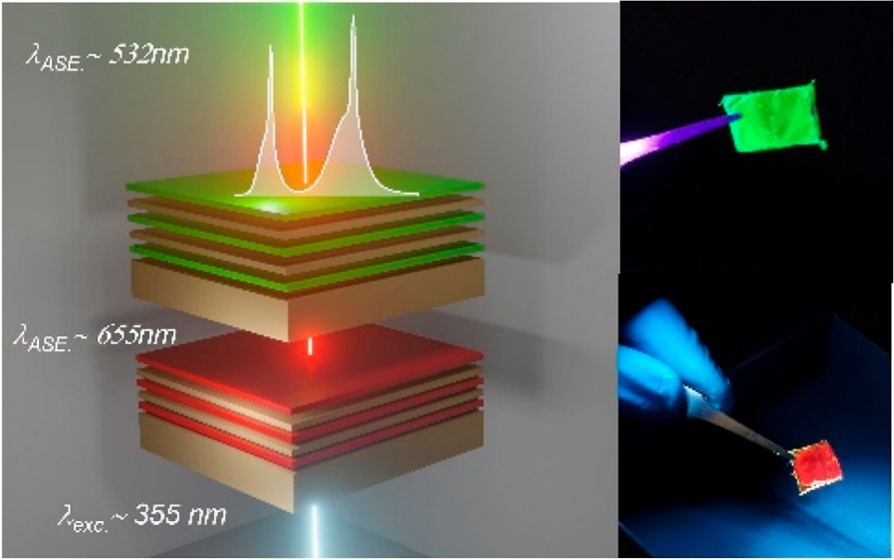

Lead halide perovskite nanocrystals (NCs) are highly suitable active media for solution-processed lasers in the visible spectrum, owing to the wide tunability of their emission from blue to red via facile ion-exchange reactions. Their outstanding optical gain properties and the suppressed nonradiative recombination losses stem from their defect-tolerant nature. In this work, we demonstrate flexible waveguides combining the transparent, bioplastic, polymer cellulose acetate with green CsPbBr_3_ or red-emitting CsPb(Br,I)_3_ NCs in simple solution-processed architectures based on polymer-NC multilayers deposited on polymer micro-slabs. Experiments and simulations indicate that the employment of the thin, free-standing membranes results in confined electrical fields, enhanced by 2 orders of magnitude compared to identical multilayer stacks deposited on conventional, rigid quartz substrates. As a result, the polymer structures exhibit improved amplified emission characteristics under nanosecond excitation, with amplified spontaneous emission (ASE) thresholds down to ∼95 μJ cm^–2^ and ∼70 μJ cm^–2^ and high net modal gain up to ∼450 and ∼630 cm^–1^ in the green and red parts of the spectrum, respectively. The optimized gain properties are accompanied by a notable improvement of the ASE operational stability due to the low thermal resistance of the substrate-less membranes and the intimate thermal contact between the polymer and the NCs. Their application potential is further highlighted by the membrane’s ability to sustain dual-color ASE in the green and red parts of the spectrum through excitation by a single UV source, activate underwater stimulated emission, and operate as efficient white light downconverters of commercial blue LEDs, producing high-quality white light emission, 115% of the NTSC color gamut.

## Introduction

Inorganic and hybrid lead halide perovskite nanocrystals (LHP NCs) have emerged as versatile, tunable, and low-cost gain media for optically pumped, solution-processed lasers in the visible spectral region. Significant advances include the demonstration of low threshold and air-stable amplified spontaneous emission (ASE) in thin films or simple waveguide structures of perovskite NCs with ambient stability of several hours and thresholds down to ∼1 μJ cm^–2^ in the femtosecond excitation regime.^1−5^ Furthermore, efficient optically pumped, single- and multi-mode LHP NC lasers have been realized based on various resonator geometries that include vertical cavities,^6−10^ distributed feedback,^11−14^ and whispering gallery architectures.^15−17^

Despite the significant progress, there are some key challenges that need to be tackled toward the realization of practical and cost-effective optically pumped amplifiers and lasers based on perovskite NCs. Improving the stability and reliability of the lasing structures operating in ambient conditions is one such pressing issue. Furthermore, the great majority of the reported work employed femtosecond laser excitation to force favorable competition of the stimulated emission process against gain losses; however, such an approach is practically problematic in terms of cost, scalability, weight, and size of the device. A more viable, compact, and cost-effective path will be the implementation of quasi-continuous wave (cw) pulsed or pure cw lasers to initiate the lasing action from the NCs. Bulk and low dimensional perovskites have been recently demonstrated to sustain ASE or lasing even under cw-excitation in properly designed structures.^11,12,18−24^ However, these results are typically obtained at cryogenic temperatures, inaccessible by commercial Peltier-type cooling, or implement rigid cavities fabricated by rather elaborate epitaxial and lithographic techniques, that limit the practicality and compromise the cost-effectiveness and mass-production promise of a solution-based fabrication methodology. Finally, the great majority of the work involves the one specific popular emitter—green-luminescent CsPbBr_3_ NCs—allowing us to thoroughly study effective ligand and encapsulation strategies. To extend the stimulated emission gamut through the visible spectrum, further work is needed to improve the lasing performance and reliability of blue and red LHP NC emitters.

Herein, we introduce a facile, fully solution-processed fabrication methodology to produce flexible waveguide structures combining cellulose acetate (CA) polymer membranes with active gain media based on multilayer stacks of CA with green CsPbBr_3_ or red-emitting CsPb(Br,I)_3_ NCs. Upon structure optimization, nanosecond excitation of such free-standing membranes results in air-stable ASE in the green and red parts of the spectrum, with low thresholds down to ∼90 and ∼70 μJ cm^–2^ and high net modal gain up to ∼450 and ∼630 cm^–1^, respectively. Reference structures produced by deposition of the same multilayer CA/NC stacks in rigid quartz substrates also exhibit ASE, but at the expense of higher thresholds and poorer ambient stability. Angle-resolved white-light reflectivity measurements combined with transfer matrix method (TMM) simulations confirm that the improved ASE threshold and net modal gain in the CA membranes are a result of better optical mode confinement that improves photon–exciton coupling. On the other hand, the absence of a bulky substrate and the less thermal dissipative CA/NC interface yields efficient heat exchange with the environment, significantly improving the operational ASE stability with respect to the reference quartz structures. The versatility and excellent gain properties of such structures allow us also to stack membranes and simultaneously sustain dual-color ASE in the green and red parts of the spectrum using a single nanosecond excitation source and operate as efficient downconverters of commercial blue LEDs, producing high-quality white light emission, 115% of the NTSC.

## Results and Discussion

### Design and Fabrication of NC-Sensitized Polymer Membranes

**Figure 1:**
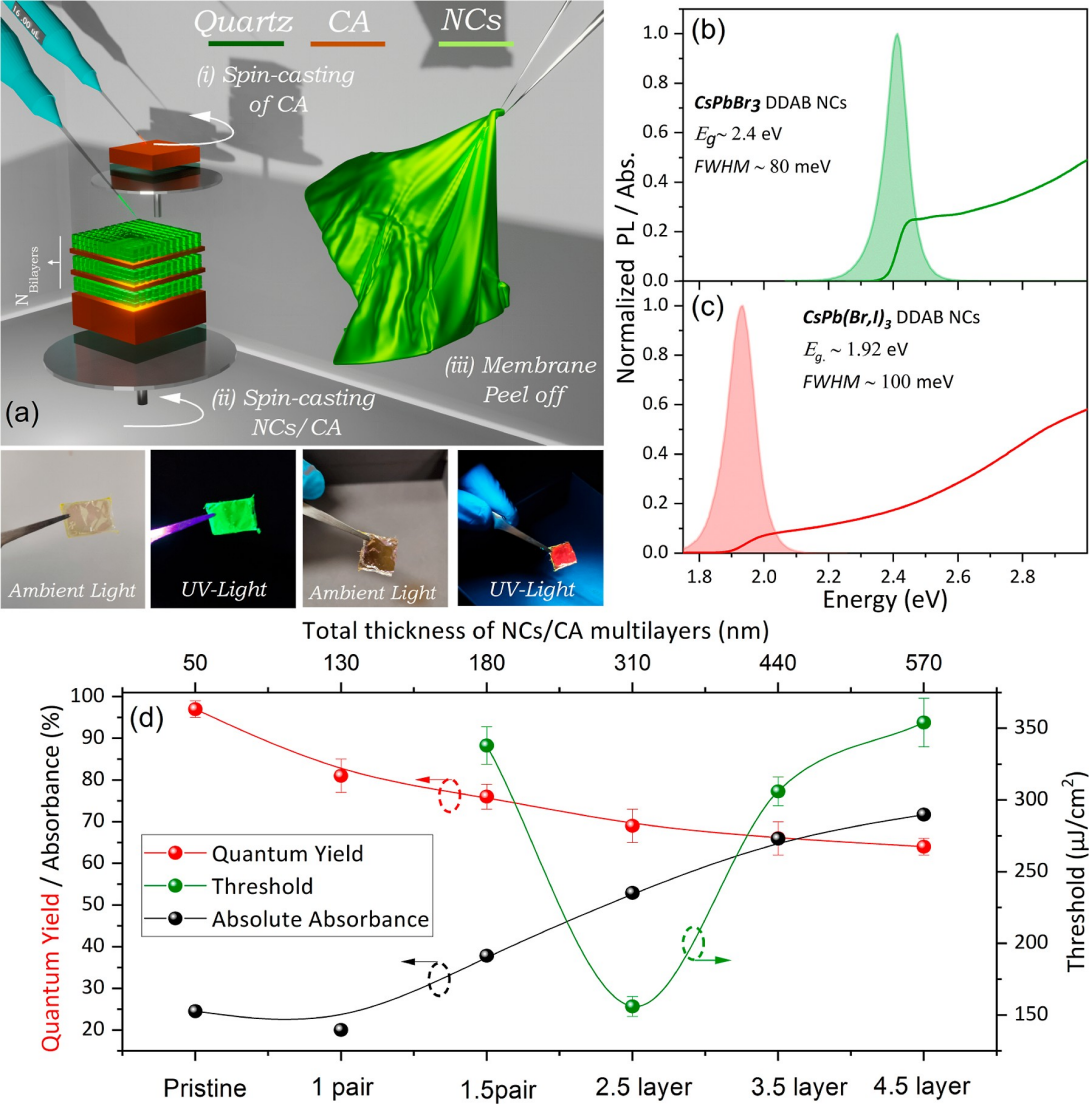
(a) Schematic of the fabrication procedure of the free-standing membranes with a multilayer of CA and NCs as the active gain media. The photos display the flexible membranes under ambient and low-power UV light irradiation. Optical absorption and PL spectra (b) CsPbBr_3_ DDAB NCs and (c) CsPb(Br,I)_3_ DDAB NC solutions. (d) Quantum yield, absorbance, and ASE threshold deposited on quartz substrate versus the overall bilayer thickness. ASE threshold data points from the pristine film and 1 pair layers are not included, as such active regions did not sustain ASE at room temperature.

The objective of the study has been the demonstration of air-stable and efficient optical amplification in the quasi-cw optical pumping regime by combining robust perovskite NC gain media with a simple, fully solution-processed, mechanically flexible cavity design. To achieve such a target, we implemented mirrorless, polymeric micro-membranes that can weakly confine an optical field due to the refractive index contrast between the organic material and the surrounding air. Free-standing membranes were fabricated by spin casting microns-thick slabs of CA onto quartz,^25^ followed by alternate deposition of multiple layers of CsPbBr_3_ or CsPb(Br,I)_3_ NCs and CA with layer thickness around 50 and 80 nm for NCs and CA, respectively. Optimization of the structural characteristics of such multilayer stacks is discussed in the following manuscript section. Upon deposition, the membranes were lifted-off from the quartz substrate via a simple peel-off technique, as schematically illustrated in Figure 1a. The figure also contains images of the NC-sensitized membranes under ambient and UV-light conditions, demonstrating their good uniformity, optical quality, and bright emission. Green emitting CsPbBr_3_ NCs with dimethyldioctadecylammonium bromide (DDAB) ligands have been selected as the active gain media, owing to their structural robustness and good photostability combined with exceptionally high photoluminescence (PL) QY approaching in the liquid phase 100% (larger than 90% in the solid state).^26^ The NCs employed were cuboids with mean sizes of ∼10 nm, as presented in the TEM images of Figure S1, centered at ∼2.40 eV with a full width half maximum (fwhm) of ∼80 meV, as shown in Figure 1b. For the red-emitting active gain media, CsPb(Br,I)_3_ NCs with variable bromine-iodine ratio were obtained via a newly designed synthesis using hot-injection methodology with oleic acid (OA) and two different branched ligands such as dioctylamine (DOAm) and DDAB. Branched ligands with shorter chains are known to have strong binding to the surface of NCs, thus they provide better surface passivation and improvement in the optical properties of these mixed halide NCs, with PL QY exceeding 80% in the liquid phase (larger than 10% in the solid state). In addition, DDA^+^ cations have a strong affinity to negative sites of cesium lead halide NCs, and Br^–^ anions from DDAB can substitute I^–^ in the structure resulting in more stable mixed CsPb(B_*x*_I_1–*x*_)_3_ NCs with an easily tunable wavelength.

The size and shape of CsPb(Br,I)_3_ NCs were confirmed by transmission electron microscopy Figure S2b, showing the presence of two populations of NCs with the average sizes of 16.86 ± 2.08 and 26.8 ± 4.23 nm. Powder X-ray diffraction (XRD) of purified CsPb(Br,I)_3_ NCs shows an orthorhombic perovskite structure (Figure S2d). Bromide and iodide atoms are well intermixed into a solid solution, giving rise to a gradual shift of XRD reflections. Their PL was centered at ∼1.92 eV with an fwhm of ∼100 neV as seen in Figure 1c.

Structures produced via single layer deposition of CsPbBr_3_ or CsPb(Br,I)_3_ NCs on quartz or CA membranes do not sustain nanosecond-excited ASE at room temperature. Instead, a multilayer approach was implemented using alternating spin-casted layers of NCs and CA, exploiting the orthogonality of the solvents of the two materials. The method enables the formation of sufficiently thick active gain regions, exceeding the cut-off thickness for the propagation of one optical mode,^27,28^ while at the same time preserving the optical quality of the layers and reducing the optical losses which single, thick casted films are prone to.^29^ Representative results of the optimization studies in such multilayers are presented in Figure 1d, where the influence of the number of CsPbBr_3_ NC/CA pairs on the light absorption, emission yield, and ASE threshold is demonstrated. The respected emission and absorption data as a function of the number of layers are displayed in Figure S3a,b. Based on such work, activation of nanosecond ASE is typically achieved for an active medium of 1.5 NC/CA layers (a sequence of ∼50 nm NC/∼80 nm spacer CA/∼50 nm NC layers), while ASE threshold minimization is obtained for a total multilayer thickness of ∼310 nm or equivalently the number of 2.5 NC/CA bilayers (a total NC thickness of ∼150 nm). The pronounced reduction of the ASE threshold in the 1.5 to 2.5 layer range is assigned to a combined effect of increased light absorption and more efficient confinement of the optical modes within the thicker active region. The effect appears to dominate over the slight reduction of the emission quantum yield observed across the same thickness range. The addition of more pairs in the multilayer stack, allows the propagation of higher-order cavity modes, effectively reducing the photon–exciton coupling. Furthermore, processes such as re-absorption and light scattering become more prominent, reducing the emission QY and suppressing the overall *Q*-factor of the cavity. Figure S3c,d contains another parametric study, probing the influence of the CA spacer layers thickness on the ASE threshold; experiments as such yielded optimum thicknesses of around 50 and 80 nm for the NC and CA layers incorporated in the multilayer stack active regions, respectively. The use of such thick CA spacer layers in the optimized waveguide structures also ensures that their optical properties are not affected by electronic interactions between the NC emitters layers.

### Room-Temperature-ASE under Nanosecond Excitation

**Figure 2:**
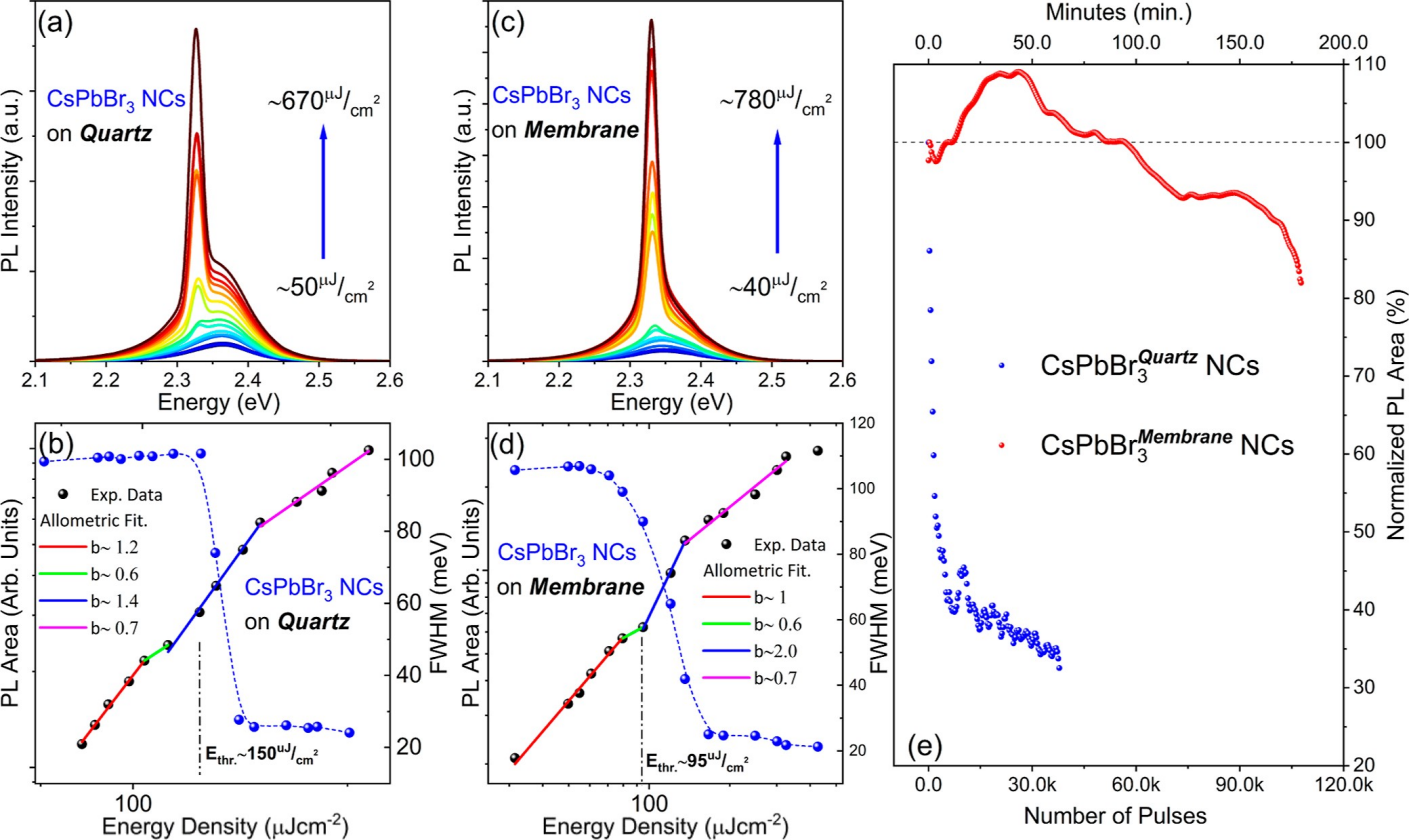
Evolution of the ASE spectra at different excitation energies for 2.5 CsPbBr_3_ NC/CA multilayers on (a) quartz and (b) CA membrane. Integrated emission and allometric fits of the data along with the emission linewidth versus excitation energy density are presented for: (c) quartz and (d) CA membrane. (e) The operational stability of ASE versus the number of excitation laser pulses.

The stimulated emission properties of CsPbBr_3_ NC/CA multilayers deposited on rigid quartz and flexible CA substrates were examined under optical excitation with a 355 nm nanosecond pulsed laser, with a pulse width of 6 ns and repetition rate of 10 Hz. Both samples exhibit a distinct transition from spontaneous emission to ASE, marked by the linear to superlinear change of slope and the simultaneous collapse of the emission linewidth from ∼100 to ∼20 meV when the pumping fluence surpasses the characteristic threshold, as seen in Figure 2a,b. The ASE thresholds were extracted via the allometric fitting of the excitation-dependent integrated areas, resulting in the champion structures displayed in Figure 2, to values of *E*_th_ ∼ 150 μJ cm^–2^ and *E*_th_ ∼ 95 μJ cm^–2^ for multilayers deposited on quartz and CA membranes, respectively. It is worth noting that ASE dominates the background spontaneous emission in both types of samples. For the multilayers on quartz, the ASE is red-shifted by ∼30 meV relative to the spontaneous emission peak, which is characteristic of the bi-excitonic optical gain mechanism.^30,31^ On the other hand, in the free-standing membrane, the ASE lies in the proximity of the PL peak as the emission couples to the Fabry Perot resonances supported by the micron-thick CA substrate. Such results are further elaborated in Figure S4, containing emission spectra from the two structures below and above the ASE threshold, as well as a comparison of emission data with TMM simulated reflectivity spectra from the CA membrane sample.

On top of sustaining ASE at consistently lower excitation fluences compared to the same gain media deposited in quartz, the polymer membranes also exhibit significantly higher stability during ASE operation. ASE stability for both types of structures was recorded under continuous nanosecond excitation at ambient conditions, at a fluence of 200 μJ cm^–2^ for illumination of up to 110 × 10^3^ laser pulses or an equivalent of 3 operational hours. Figure 2e contains the results of such a study performed on the same champion devices, the ASE data of which are presented in Figure 2a–d. For the CA membrane structure, the ASE intensity exhibited an initial increase of 10% up at ∼25 × 10^3^ laser pulses, followed by a gradual emission quenching down to 85% of the initial intensity, which is a loss of only 15% after 3 h of operation. The initial increase of the ASE intensity was observed also in photostability tests of other free-standing membrane samples and it could be related to the self-healing and annealing effects of the NCs, during the intense photo-excitation.^32,33^ In contrast, the multilayers deposited on quartz exhibit a rapid loss of the output intensity, with complete quenching of the ASE at ∼40 × 10^3^ pulses or equivalently 1 h of operation.

The improved ASE stability in the free-standing membranes is predominantly ascribed to the more efficient heat transfer and the smaller thermal interface resistance of NCs/CA compared to the respective heat conduction and heat dissipation properties of the structures deposited on quartz. Heat extraction through the substrate depends on the material thermal conductivity and the thickness; even though the thermal conductivity of CA (∼0.17 W m^–1^K^–1^)^34^ is 1 order of magnitude smaller than quartz (∼1.6 W m^–1^K^–1^),^35^ the CA membranes are more than 300 times thinner than the conventional 1.1 mm thick quartz substrates employed in our studies, allowing a more efficient heat flow through the CA substrate. Furthermore, the CA polymer is conformable and can displace more of the air entrapped in the CA–NC interfaces than the rigid quartz-NC interface, reducing the thermal interface resistance and, thus, the heat accumulated in the material heterointerface. Further evidence for such an explanation can be found in Figure S5, in which the ASE peak position is monitored as a function of the number of excitation pulses. For the multilayers deposited on quartz, an appreciable ∼20 meV blue shift of the ASE builds up during the one-hour operation lifetime of the structure. The blue shift can be predominantly attributed to NC heating arising from the anomalous temperature-dependent variation of the energy gap in CsPbBr_3_ NCs.^36^ In contrast, the ASE peak position in the free-standing membrane is considerably more stable, showing a much smaller ∼7 meV red-shift over the course of the 3 h operation. Such a bathochromic shift may originate from the thermal expansion of the CA substrate and/or a small degree of NC sintering during the intense laser illumination.^37^ Overall, in all membrane samples, the ASE peak position exhibited a small red or blue shift of the order of 5 to 10 mV, being always significantly smaller in magnitude compared to the 20 to 30 meV blue shift of the ASE peak in quartz samples, confirming the improved thermal properties of the former structures.

**Figure 3:**
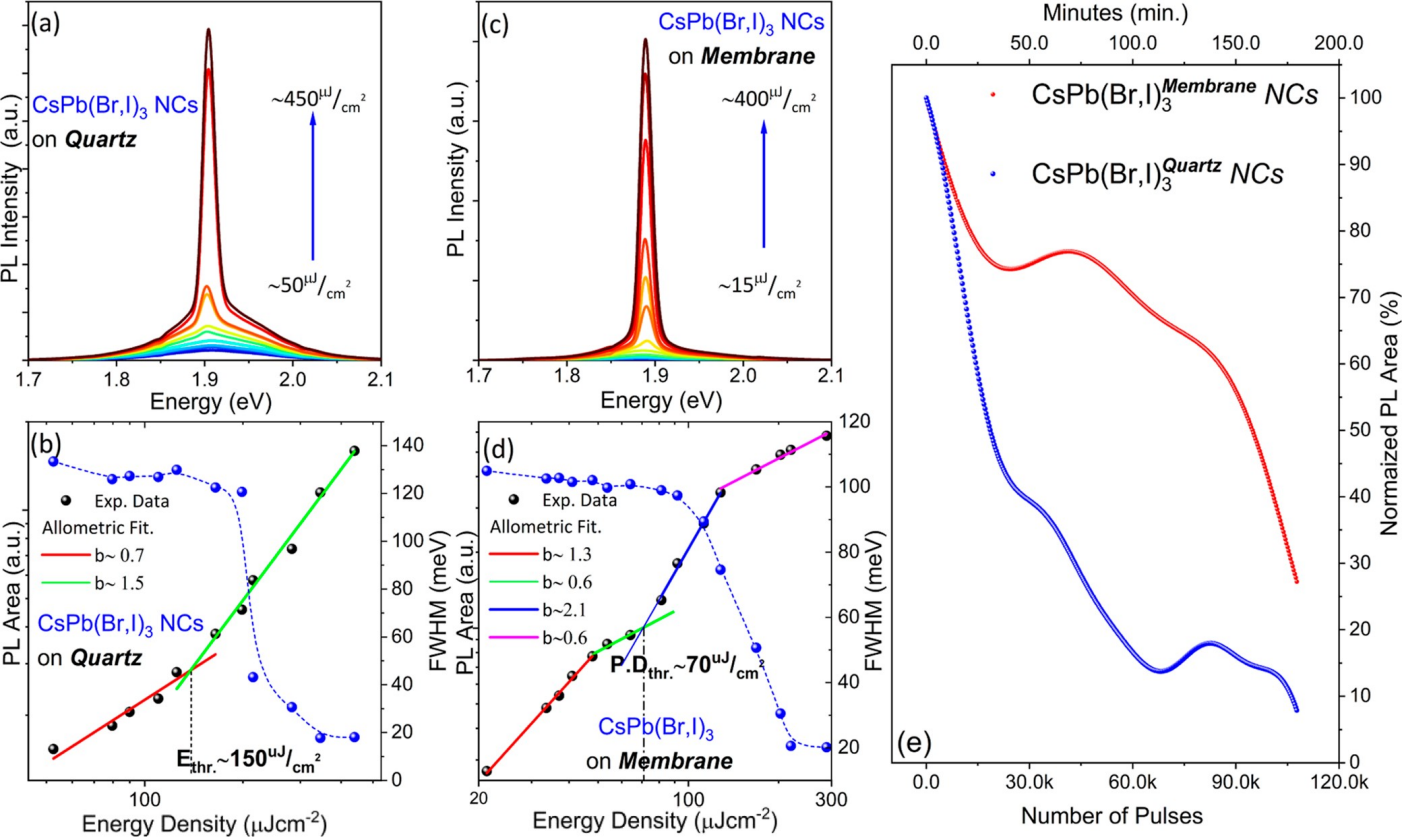
Excitation-dependent emission spectra at different excitation energies for CsPb(Br,I)_3_/CA multilayers on (a) quartz and (b) CA membrane. Integrated PL area with the allometric fits of the data along with the emission linewidth versus excitation energy density for (c) quartz and (d) CA membrane. (e) Operational stability of ASE versus the number of excitation laser pulses.

Following an identical fabrication procedure, red-emitting CsPb(Br,I)_3_ NC/CA multilayers were also produced on quartz and free-standing membranes, examined under optical excitation with a 532 nm nanosecond pulsed laser, with a pulse width of 6 ns and repetition rate of 10 Hz. Similar to the CsPbBr_3_ NC-based structures, the CsPb(Br,I)_3_ NC sensitized membranes exhibit consistently lower ASE threshold and higher output stability compared to the respected samples on quartz. The main differences relative to the green-emitting analogous structures are that on the average: (i) the ASE threshold reduces even more in the flexible structures, that is from ∼150 μJ cm^–2^ in quartz to ∼70 μJ cm^–2^ in CA for the champion structures data shown in Figure 3a–d; (ii) the improvement in operational stability is substantial but smaller than that in the green-emitting samples as observed in Figure 3e. It is worth noting that to the best of our knowledge the aforementioned ASE threshold of ∼70 μJ cm^–2^ constitutes a record low value for red-emitting perovskite NC structures in the nanosecond excitation regime.^30,38,39^

The improved ASE stability can again be attributed to the superior heat transfer properties of the flexible membranes. This is evidenced by experiments summarized in Figure S6 that account for the local heating by monitoring the ASE position shift and further confirmed by the temperature and thermal images of the samples obtained by an IR camera shown in Figure S7. It can be observed that during the first 7 min of ASE operation, the local temperature on the quartz deposited waveguides exhibits a temperature rise of ∼1.35 °C, which is ∼3.5 times higher than the respective temperature rise of the polymer membrane. Furthermore, as presented in Figure S8, the free-standing membranes can sustain ASE under UV (355 nm) nanosecond excitation, albeit at increased threshold values compared to the respected values using green (532 nm) excitation. Importantly though no ASE was observed under 355 nm pumping from multilayer CsPb(Br,I)_3_ NC structures deposited on quartz, indicative of the better waveguiding and photostability properties of the free-standing membranes, as elaborated further by optical gain measurements and TMM simulations, discussed below.

### Optical Gain Measurements

**Figure 4:**
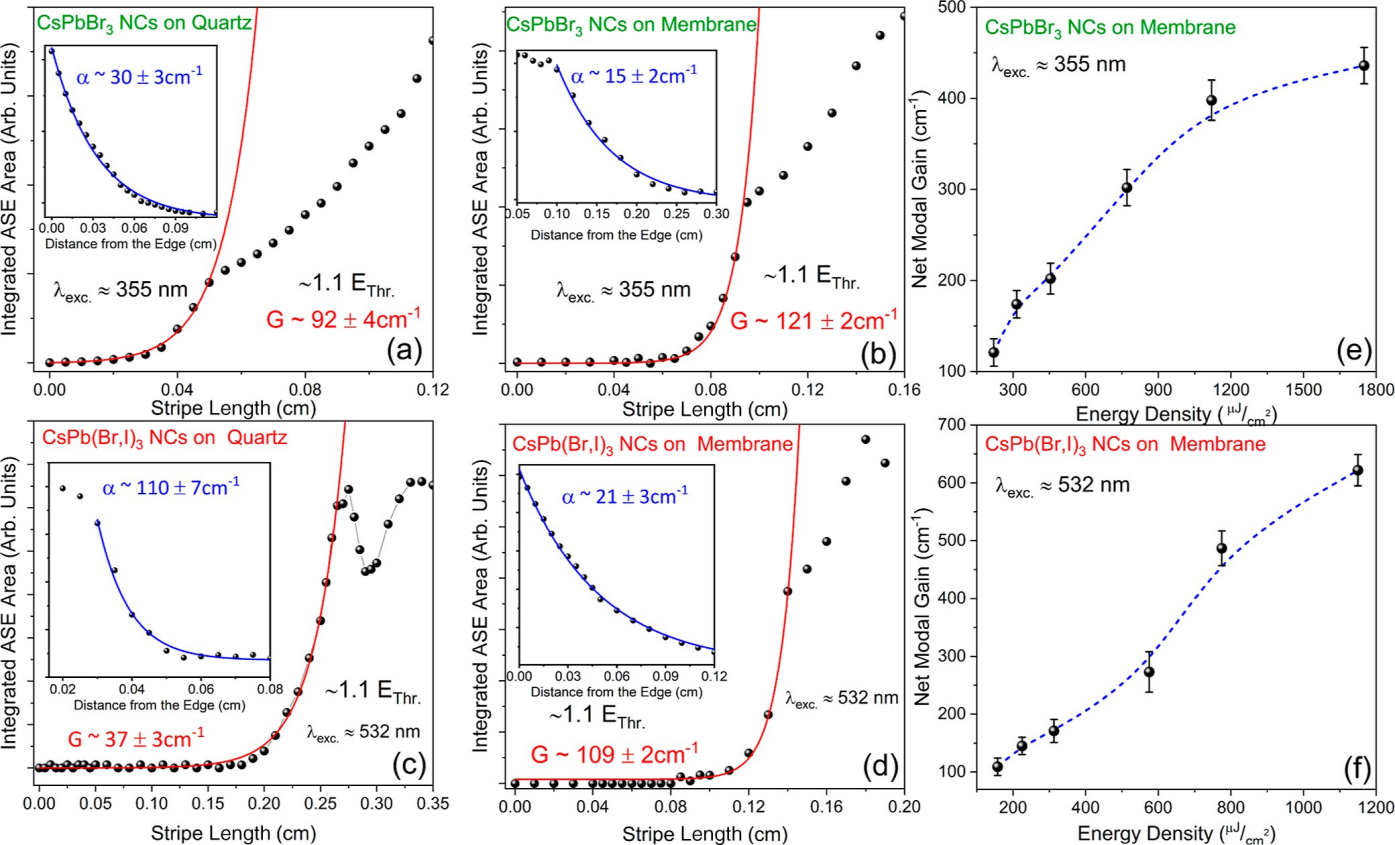
Integrated emission intensity versus light stripe length obtained using VSL experiments for multilayers of: (a) CsPbBr_3_ NC/CA on quartz, (b) CsPbBr_3_ NC/CA on CA membrane, (c) CsPb(Br,I)_3_ NC/CA on quartz, and (d) CsPb(Br,I)_3_ NC/CA on CA membrane. The inset figures display the results of the optical loss as a function of the stripe distance from the edge of the sample, fitted via Beer–Lambert law type of curves. Net modal gain coefficients as a function of the excitation energy density for (e) for CsPbBr_3_ multilayers and (f) CsPb(Br,I)_3_ multilayers on CA slabs.

The optical gain and waveguiding properties of the green and red-emitting NC multilayers on quartz and free-standing membranes were further investigated via variable stripe length (VSL) experiments.^30,40−42^ A line-shaped excitation beam was focused onto the sample surface via a cylindrical lens. At the same time, the ASE emission was collected from the edge of the sample as a function of stripe length, enabling the estimation of the net modal gain *G*. The optical loss, α, related to re-absorption and scattering losses within the structure was evaluated using a fixed stripe length beam by varying the distance of the light stripe from the edge of the sample. The results of the aforementioned VSL experiments at an excitation of ∼1.1 *E*_th_ (ASE threshold) are presented in Figure 4a–d for red and green-emitting multilayer stacks deposited on quartz and polymer substrates.

The ASE onset is characterized by the steep increase of the integrated emission intensity when the stripe size reaches the required length for the optical gain to surpass the propagation losses. To estimate the net modal gain, the experimental data were fitted using eq 1, near the threshold region.^30,40^
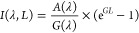
1Where *A*(λ) = η*g*(λ), η denotes the cross-sectional geometrical factor, g is the optical gain of the material, and *L* is the excitation stripe length.^43^ For green-emitting CsPbBr_3_ NC-based active media, the net modal gain *G* is estimated at ∼92 ± 4 and ∼121 ± 2 cm^–1^, for quartz and free-standing membranes, respectively. Lower net modal gain values of ∼37 ± 3 and ∼109 ± 2 cm^–1^ were obtained for mixed halide CsPb(Br,I)_3_ NC-based structures on quartz and CA, respectively.

The correlation of optical gain, loss, and net modal gain is given by eq 2(30,40)
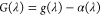
2

The absorption loss α is extracted by the fitting of the integrated emission versus excitation strip distance from the edge of the sample using the following Beer–Lambert law function^40^
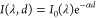
3Where *I*_0_ is the integrated emission for *d* = 0 and *d* corresponds to the distance of the excitation stripe from the sample edge. The fitting results are presented in the inset graphs of each sample in Figure 4a–d. As can be seen, a substantial reduction of the absorption losses by a factor of ∼2 and ∼5 for green and red-emitting structures is observed when switching from quartz to free-standing membranes. As re-absorption losses in the rigid and flexible slabs are expected to be similar due to the identical characteristics of the multilayer active region, the reduction of the optical losses can be attributed to a suppression of scattering losses in the free-standing membrane as the emission is more efficiently waveguided through the optical modes supported by the CA slab. The effect of pump excitation energy on the net modal gain *G* for the green and red-emitting flexible waveguides, is presented in Figure 4e,f. For both structures, the net modal gain grows with pump fluence to values as high as ∼430 cm^–1^ at 1.7 mJ cm^–1^ and ∼620 cm^–1^ at 1.2 mJ cm^–1^ for CsPbBr_3_ and CsPb(Br,I)_3_ NC-based emitters. The lower *G* values and the smaller rate with which the net modal gain grows with fluence in the green-emitting samples are attributed to the higher optical losses, such as the Auger and photocharging processes, associated with the UV (355 nm) photoexcitation compared to the green (532 nm) excitation used of the mixed-halide NC-based structures. A summary of the ASE and optical gain properties of the studied samples is listed in Table 1.

**Table 1:** ASE Threshold and Optical Gain Properties of the Studied Samples^a^

	sample	*E*_th_ (μJ cm^–2^) λ_exc._ ∼ 355 nm	*E*_th_ (μJ cm^–2^) λ_exc._ ∼ 532 nm	α (cm^–1^) @1.1 *E*_th_	*G* (cm^–1^) @1.1 *E*_th_	*g* (cm^–1^) @1.1 *E*_th_	*G* (cm^–1^) @16 *E*_th_
quartz	CsPbBr_3_ NC multilayers	150		30	92	122	
	CsPb(Br,I)_3_ NC multilayers		150	110	37	147	
membranes	CsPbBr_3_ NC multilayers	95		15	121	137	430
	CsPb(Br,I)_3_ NC multilayers	300	70	21	109	130	620

a The missing data in the first two table columns, refer to samples in which no ASE was observed under the specified experimental conditions. The missing data in the last column, refer to samples in which ASE was not sustained at such high excitation.

### Waveguiding Properties of the Membrane Structures

**Figure 5:**
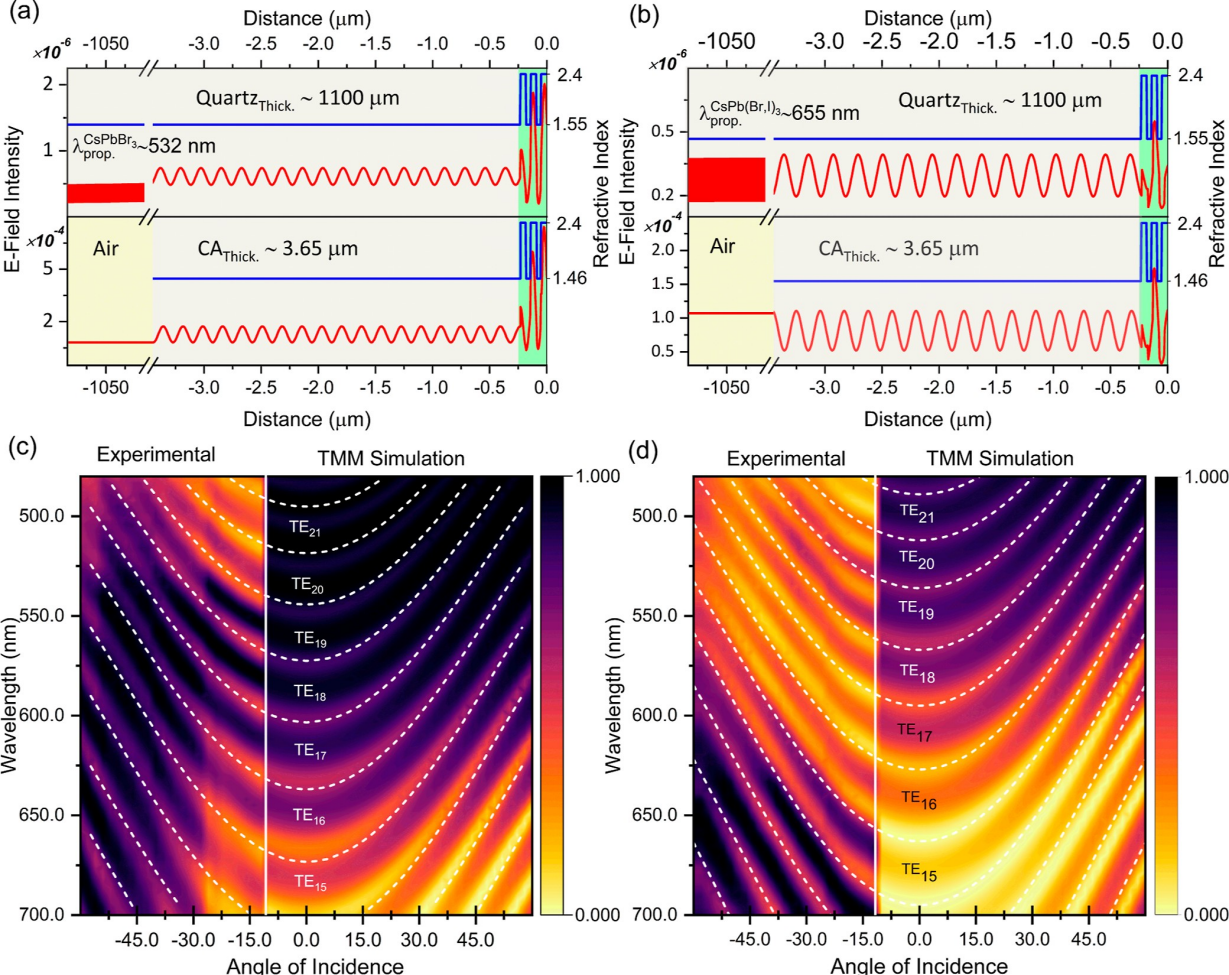
Cross-sectional electric field distribution on quartz and free-standing membranes for (a) CsPbBr_3_ and (b) CsPb(Br,I)_3_ NC/CA multilayers. Angle-dependent white light reflectivity and TMM simulation data of the polymer waveguides containing (c) CsPbBr_3_ and (d) CsPb(Br,I)_3_ NC/CA multilayers. The white dashed lines represent the dispersion of the optical modes, while the continuous straight light separates experimental and model data.

The TMM model was used to calculate the cross-sectional electric field distribution within the green- and red-emitting waveguide structures deposited in CA and quartz. The data are presented in Figure 5a,b, along with the refractive index modulation and the layer thicknesses. The striking observation from the simulation data is that the electric field intensity is 2 orders of magnitude larger in the membrane slabs compared to the quartz deposited structures. This is largely a result of the significantly smaller CA slab thickness that effectively confines the optical field within the structure. A more careful look into the model data indicates that the emission of the CsPbBr_3_ and CsPb(Br,I)_3_ NC-based structures couples to the 20th and 15th cavity mode, respectively. This confirms that the reduction of the ASE threshold and the simultaneous increase of the net modal gain observed in the membrane samples can be attributed to such efficient light waveguiding properties.

Angle-dependent white-light reflectivity performed on the structures further confirms the formation of dispersive resonances, corresponding to different order cavity modes, as seen in Figure 5c,d. Experimental data show good agreement with simulated data produced via a TMM model. Based on the reflectivity results, quality factors (*Q* ∼ λ/Δλ) of ∼28 and ∼31 were estimated for the green and red-emitting cavities, respectively that are comparable with *Q*-factors obtained in dielectric slab microcavities formed electron-beam evaporation of conventional inorganic dielectric materials.^44^

### Dual-Color ASE and White Light Generation

Lasers that can produce dual or multicolor emission toward white light lasing can find niche applications in full-color laser lighting/imaging/displays, spectroscopy, fluorescence sensing, and visible light communications.^45−51^ Monolithic integration of RGB laser multilayers based on epitaxial semiconductors is challenging though, due to the large lattice mismatch involved. Fabrication of single-chip, multicolor lasers based on soft, solution-processed semiconductor gain media such as polymers, nanowires, or NCs can circumvent the lattice mismatch issue, yet further work is needed toward improvements in the design, capabilities, and reliability of such laser devices. Toward such an objective, a preliminary study of dual ASE emission was performed by optical excitation of stacked up green-emitting CsPbBr_3_ and red-emitting CsPb(BrI)_3_ NC-based membranes.

**Figure 6:**
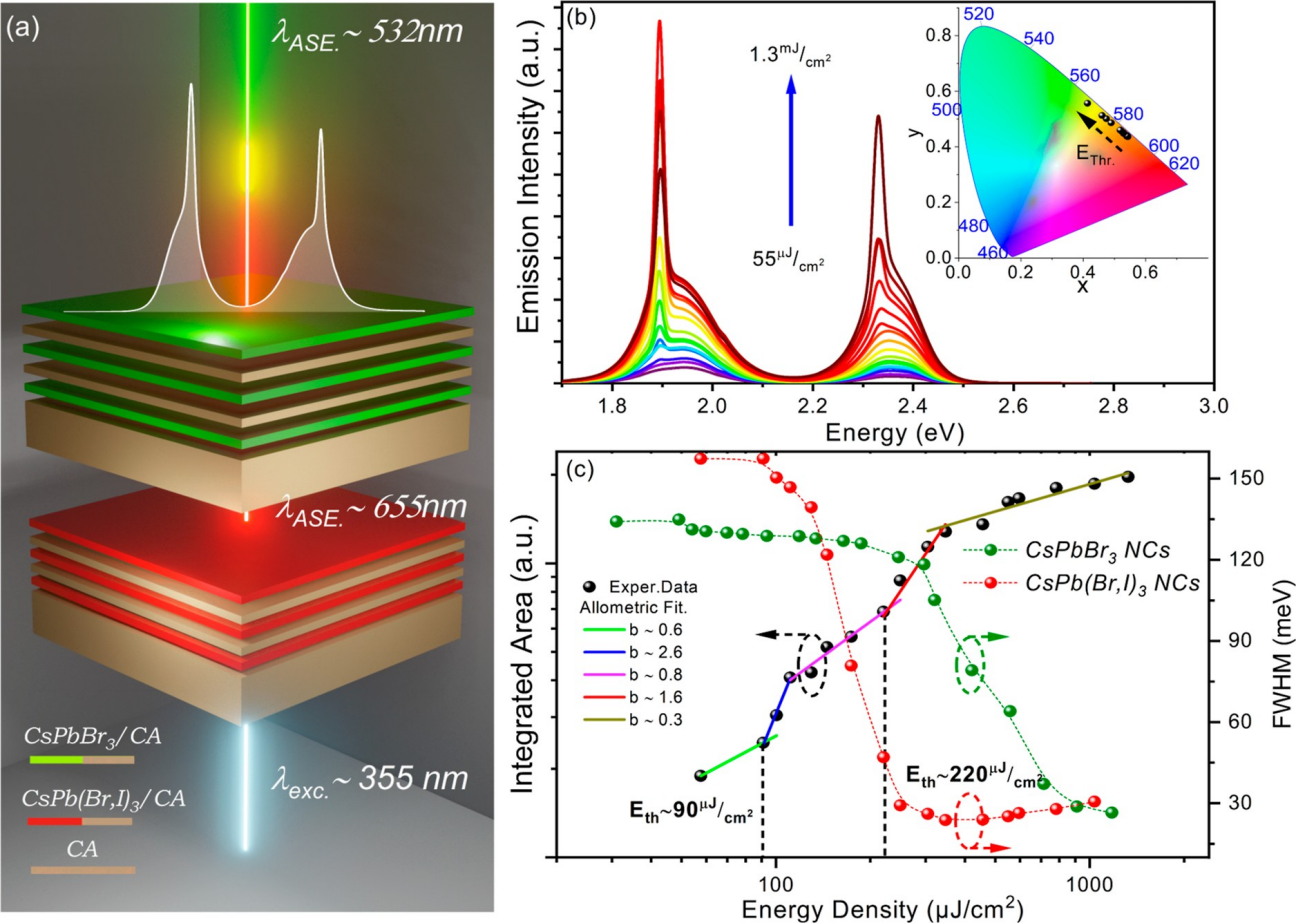
(a) Schematic illustration of the geometry used to initiate dual ASE from stacked red- and green-emitting free-standing membranes. (b) Evolution of the emission spectra at different pump fluences for stacks of CsPb(Br,I)_3_/CsPbBr_3_—CA multilayers demonstrating their ability to sustain dual-color ASE. The inset CIE diagram indicates the tunability of the ASE emission chromaticity via the excitation fluence. (c) Integrated area of the combined emission from the stacked membranes and fwhm of the emission of each of the two membranes plotted against excitation energy density.

Optical pumping of the stacked membranes was performed via 355 nm nanosecond pulses, exciting first the CsPb(BrI)_3_ NC-based structure, as shown in the schematic of Figure 6a, to minimize reabsorption losses of the green ASE peak by the red-emitting overlayer membrane. The spectral evolution as a function of excitation fluence is presented in Figure 6b, while the combined integrated ASE emission from the stacked membranes and the fwhm of each of the two ASE peaks is plotted against excitation energy density, as shown in Figure 6c. Red ASE is activated at ∼90 μJ cm^–2^ while green ASE is switched on at ∼220 μJ cm^–2^ with the dual ASE peaks sustained to densities larger than 1 mJ cm^-2^. The far-field mixing of the multicolor ASE is displayed on a CIE color map, highlighting the ability to tune the color chromaticity of the output emission from red to green by adjusting the excitation intensity.

**Figure 7:**
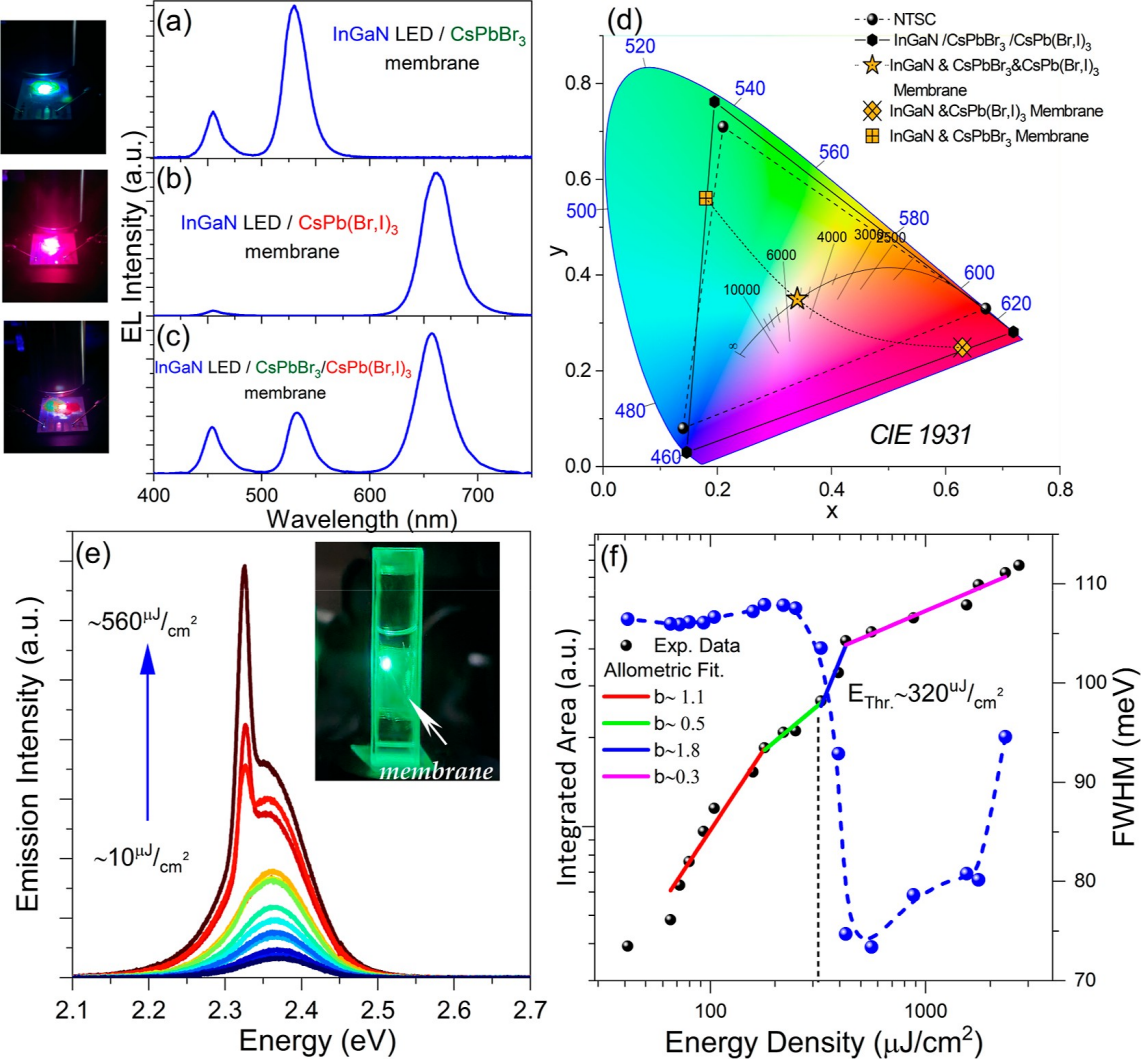
Optical spectrum of combined blue-emitting InGaN LEDs with (a) CsPbBr_3_ NC, (b) CsPb(Br,I)_3_ NC, and (c) combination of both membranes. The inset photos show the hybrid LEDs under operation. (d) Color gamut of the hybrid white-light system according to CIE 1931 color space in comparison with the National television system committee (NTSC) standard. (e) Evolution of ASE spectra of CsPbBr_3_ membranes immersed in deionized (DI) water. The inset photo shows the membrane under operation. (f) Integrated emission and spectral linewidth as a function of the excitation fluence.

The potential of the NC-sensitized free-standing membranes for other applications such as light downconverters for white light generation and underwater light communication was also explored, with blue-green lasers being considered ideal candidates, to satisfy the demand for high-bandwidth, high-speed wireless communication due to the large range data transfer with low propagation losses in aqueous environments. For the former function, green and red-emitting membranes were individually or jointly integrated with blue-emitting InGaN LEDs. As presented in Figure 7a–c, the blue electroluminescence can be partially converted to green or red emission by respected combinations of the InGaN diodes with green or red emissive NC/CA multilayers. Integration of the green and red membranes to the nitride LEDs results in RGB emission. By exploiting the fact that the emission chromaticity can be finely tuned via the LED driving current, a pure white color of CIE coordinates 0.34, 0.35 at a color temperature of ∼5000 K, and an NTSC 115% wide color gamut can be demonstrated, as shown in Figures 7d and S9. The results indicate that such lightweight, thin, and bendable structures are promising for applications in flexible lighting panels and displays.

The small solubility of CA in DI water combined with encapsulation of the flexible membranes by the Hyflon AD 60 fluoropolymer,^52^ enabled the demonstration of nanosecond-excited ASE in an aqueous environment, as presented in Figure 7e. Upon UV-excitation of CsPbBr_3_ NC-based structures, an increase of fluence results in the activation of ASE at ∼2.33 eV with an ASE onset at *P*_th_ ∼ 320 μJ cm^–2^, as presented in Figure 7f. The increase of the threshold by a factor of ∼3, compared to the respective samples investigated in ambient conditions, can be assigned to the reduction of the refractive index contrast between the membrane (*n*_CA_ ∼ 1.46) and the surrounding medium (*n*_DI water_ ∼ 1.33 vs *n*_air_ ∼ 1) resulting in higher optical losses of the resonator as well as the degradation of the water-soaked structure.

## Conclusions

In summary, we have demonstrated the facile, fully solution-processed fabrication of flexible, free-standing structures, combining CA polymer membranes with active gain media based on multilayer stacks of CA with green CsPbBr_3_ or red-emitting CsPb(Br,I)_3_ NCs. Such flexible membranes act as low *Q* waveguides supporting optical modes that can effectively couple with the perovskite gain media emission resulting in nanosecond-excited ASE with a lower threshold, higher net modal gain, and suppressed optical losses compared to identical multilayers deposited on rigid quartz substrates. Furthermore, they exhibit higher ASE operational stability compared to the latter samples as a result of the better heat outflow properties arising from the absence of a supporting substrate and the smaller thermal interface resistance between the two soft and comfortable CA and NC media. The exceptional optical amplification properties of such membranes are witnessed by their ability to stack up and sustain dual-color ASE in the green and red parts of the spectrum, while their versatility for other photonic applications is confirmed by demonstrations of white light generation and underwater ASE. The presented results demonstrate the high potential of simple, solution-processed waveguides based on polymer resonators and LHP NC gain media for practical, scalable, flexible, and low-cost spontaneous and stimulated emission applications.

## Materials and Methods

### NC Synthesis

#### CsPbBr_3_ DDAB NCs

The synthesis of CsPbBr_3_ DDAB NCs is similar to the method described in ref (53).

#### CsPb(Br,I)_3_ DDAB NCs

##### Preparation of Cesium Oleate Precursor Solution

(Cs-oleate, 0.4 M). Cs_2_CO_3_ (814 mg, 2.5 mmol) was mixed with octadecene (10 mL) and OA (2 mL, 6.34 mmol) in a vial. The mixture was stirred and heated to ca. 125 °C on a hot plate until it became clear and then cooled to room temperature.

##### Synthesis of CsPb(Br,I)_3_ NCs

PbI_2_ (200 mg, 0.434 mmol) and DDAB (92.5 mg, 0.2 mmol) were added to 10 mL mesitylene in a 100 mL 3-necked flask. The mixture was heated to 110 °C under a nitrogen atmosphere and. After the reaction mixture reached set temperature, OA (1 mL, 3.17 mmol) and DOAm (2 mL, 6.63 mmol) were injected simultaneously. Then the temperature of the mixture was raised to 140 °C forming a clear yellowish solution, and then, the Cs-oleate (0.3 mL, 0.12 mmol) precursor was swiftly injected. After 10s the reaction mixture was cooled down to RT with a water/ice bath.

##### Isolation and Purification of CsPb(Br,I)_3_ NCs

The purification procedure was performed in the nitrogen glove box using anhydrous solvents. The crude solution was centrifuged at 5000 rpm (3438*g*) for 7 min. The supernatant was discarded, and the precipitate was dispersed in 10 mL of anhydrous hexane and then centrifuged at 3700 rpm (1883*g*) for 2 min. The precipitate was discarded, and 7 mL of anhydrous methyl acetate was added to the supernatant, followed by centrifugation at 10,000 rpm (13751*g*) for 5 min. The resulting precipitate was dispersed in 3 mL anhydrous cyclohexane, followed by centrifugation at 3700 rpm (1883*g*) for 2 min. The precipitate was discarded, and the obtained colloidal solution of CsPb(Br/I)_3_ NCs was filtered with a PTFE-syringe filter (pore size 0.2 μm) and used for further studies.

### Sample Preparation

Quartz substrates with dimensions of 2 × 1.5 cm were used for the deposition of the NC/CA multilayers and the CA slabs. Prior to the deposition process, the substrates underwent sequential cleaning with *n*-butyl acetate, acetone, and IPA. The substrates were then kept in nitric acid for 8 h allowing them to form a highly hydrophilic surface. The samples were then washed with deionized water, followed by sequential cleaning with *n*-butyl acetate, acetone, and IPA and dried under compressed air.

### Flexible Membranes

CA (CA, Mn = 30,000, η_ca_ ∼ 1.46) dissolved in acetone at 120 mg/mL was prepared for the formation of free-standing and spacer layers used in the samples. For the formation of the free-standing membranes, cellulose acetate was spin cast under static conditions on a quartz substrate at 4000 rpm for 30 s. The thickness of the free-standing membranes was determined via the single-point ellipsometry method, measured by a ThetaMetrisis ellipsometer (FR-PRO).

### LHP NCs/CA Multilayers

Multilayers were deposited either directly on top of quartz substrates or to the CA flexible substrates via spin casting. Initially, CsPbBr_3_ or CsPb(Br,I)_3_ NCs dispersed in toluene (∼30 mg/mL) was deposited under static coating conditions, at 1000 rpm for 30 s, followed by a drying step at 4000 rpm for 10 sec. CA diluted in acetone (13 mg/mL), was used as the spacer layer deposited via dynamic deposition of CA at 5000 rpm. The bilayer deposition was repeated as many times as needed to produce multilayers. In the case of the membranes, subsequent to multilayer deposition, they were peeled off from the substrate.

### TMM Model

A TMM model was used to simulate the reflectivity from the studied waveguides as well as produce the cross-sectional distribution of the electric field within the structures. The TMM model is described in detail in the book “Basics of Optics of Multilayer Systems”.^54^ The absorption spectra of the CsPbBr_3_ and CsPb(Br,I)_3_ NCs were used as the input of the TMM model, with the amplitude of the excitonic absorption corresponding to the oscillator strength of the excitonic species.

### Optical Spectroscopy

#### Steady-State PL and Optical Absorbance

The absorbance of films was acquired using a PerkinElmer Lamda 1050 spectrophotometer with a spectra range of 200–3000 nm. Steady-state PL was excited by a 405 nm diode laser and detected via a combination of a 0.75 m Acton 750i Princeton spectrometer and a 1024 × 256 pixels PIXIS charge-coupled device (CCD) camera.

### White-Light Angle-Resolved Reflectivity

A custom-made goniometer was used, equipped with a fiber-coupled tungsten-halogen white light source coupled to a 600 μm core multimode fiber. The white light beam was focused onto the sample’s surface using a collimating and a focusing lens mount on the excitation arm, in a 2 mm spot diameter. A second rotating arm was then used to collect the reflected light via a fiber-coupled Ocean-Optics CCD spectrometer, with a spectral resolution of ∼1 nm.

### Amplified Spontaneous Emission Experiments

The optical excitation of the films was performed via Quantel Brilliant Nd/YAG laser either 355 or 532 nm wavelength, producing a 4 mm beam with a pulse width of ∼6 ns and repetition rate of 10 Hz. The excitation energy density was varied via neutral density optical filters. The excitation was performed using a line-shaped beam, focused on the sample via a cylindrical lens. The PL and ASE were collected from the sample side using a Mitutoyo 10×, 0.28 NA long working distance objective coupled to a 100 μm core multimode fiber. The collected emission spectra were dispersed in a 0.75 m Acton 750i Princeton spectrometer equipped with a 1024 × 256 pixels PIXIS CCD camera.

### Optical Gain Properties

For the estimation of the optical gain properties of our samples, VSL experiments were used. The excitation beam was focused via a cylindrical lens in a 5 × 1 mm spot. The length of the strip was adjusted via a precision-adjustable slit with a micrometer sensitivity of 5 μm. For the optical loss measurements, the excitation beam was initially placed at the edge of the sample. By keeping the excitation beam size, intensity, and position constant, the sample was moved in 50 μm intervals away from the light collection side.

### Transmission Electron Microscopy

TEM images were collected using a JEOL JEM2200FS microscope operating at 200 kV accelerating voltage.

## Supporting Information Available

TEM images of CsPbBr_3_ NCs; synthetic procedure and structural characterization of CsPb(Br,I)_3_ NCs; optimization of the ASE threshold based on multilayers of CsPbBr_3_ NCs-CA on quartz substrates; ASE data and simulated reflectivity spectra from of CsPbBr_3_ NC multilayers deposited on quartz and CA; ASE stability of CsPbBr_3_ and CsPb(Br,I)_3_ NCs on quartz and membrane substrates; thermal images of bilayers deposited on CA membranes and quartz substrates; ASE studies under nanosecond UV (355 nm) excitation; light downconversion properties of NC-sensitized polymer membranes (PDF)

## References

[ref1] Zhang L.; Yuan F.; Dong H.; Jiao B.; Zhang W.; Hou X.; Wang S.; Gong Q.; Wu Z. One-Step Co-Evaporation of All-Inorganic Perovskite Thin Films with Room-Temperature Ultralow Amplified Spontaneous Emission Threshold and Air Stability. ACS Appl. Mater. Interfaces 2018, 10, 40661–40671. 10.1021/ACSAMI.8B15962/SUPPL_FILE/AM8B15962_SI_001.PDF. 30394084

[ref2] Wang Y.; Zhi M.; Chang Y.-Q.; Zhang J.-P.; Chan Y. Stable, Ultralow Threshold Amplified Spontaneous Emission from CsPbBr3 Nanoparticles Exhibiting Trion Gain. Nano Lett. 2018, 18, 4976–4984. 10.1021/ACS.NANOLETT.8B01817/SUPPL_FILE/NL8B01817_SI_001.PDF. 30011210

[ref3] Tong Y.; Bladt E.; Aygüler M. F.; Manzi A.; Milowska K. Z.; Hintermayr V. A.; Docampo P.; Bals S.; Urban A. S.; Polavarapu L.; Feldmann J.; Tong Y.; Manzi A.; Hintermayr V. A.; S Urban D. A.; Polavarapu D.; eldmann D. F.; rban D. S.; eldmann D.; Bladt E.; Aygüler M. F.; rof DrP Docampo P.; ZMilowska D. Highly Luminescent Cesium Lead Halide Perovskite Nanocrystals with Tunable Composition and Thickness by Ultrasonication. Angew. Chem., Int. Ed. 2016, 55, 13887–13892. 10.1002/ANIE.201605909. 27690323

[ref4] Krieg F.; Ochsenbein S. T.; Yakunin S.; Ten Brinck S.; Aellen P.; Süess A.; Clerc B.; Guggisberg D.; Nazarenko O.; Shynkarenko Y.; Kumar S.; Shih C.-J.; Infante I.; Kovalenko M. V. Colloidal CsPbX3 (X = Cl, Br, I) Nanocrystals 2.0: Zwitterionic Capping Ligands for Improved Durability and Stability. ACS Energy Lett. 2018, 3, 641–646. 10.1021/ACSENERGYLETT.8B00035/SUPPL_FILE/NZ8B00035_LIVESLIDES.MP4. 29552638 PMC5848145

[ref5] Imran M.; Caligiuri V.; Wang M.; Goldoni L.; Prato M.; Krahne R.; De Trizio L.; Manna L. Benzoyl Halides as Alternative Precursors for the Colloidal Synthesis of Lead-Based Halide Perovskite Nanocrystals. J. Am. Chem. Soc. 2018, 140, 2656–2664. 10.1021/JACS.7B13477/SUPPL_FILE/JA7B13477_SI_001.PDF. 29378131 PMC5908184

[ref6] Zhao C.; Tao J.; Tian J.; Weng G.; Liu H.; Liu Y.; Yan J.; Chen S.; Pan Y.; Hu X.; Chen S.; Akiyama H.; Chu J. High Performance Single-Mode Vertical Cavity Surface Emitting Lasers Based on CsPbBr3 Nanocrystals with Simplified Processing. Chem. Eng. J. 2021, 420, 127660 10.1016/J.CEJ.2020.127660.

[ref7] Huang C.-Y.; Zou C.; Mao C.; Corp K. L.; Yao Y.-C.; Lee Y.-J.; Schlenker C. W.; Jen A. K. Y.; Lin L. Y. CsPbBr3 Perovskite Quantum Dot Vertical Cavity Lasers with Low Threshold and High Stability. ACS Photonics 2017, 4, 2281–2289. 10.1021/ACSPHOTONICS.7B00520/SUPPL_FILE/PH7B00520_SI_001.PDF.

[ref8] Zhang Q.; Shang Q.; Su R.; Do T. T. H.; Xiong Q. Halide Perovskite Semiconductor Lasers: Materials, Cavity Design, and Low Threshold. Nano Lett. 2021, 21, 1903–1914. 10.1021/ACS.NANOLETT.0C03593. 33435686

[ref9] Wang Y.; Li X.; Nalla V.; Zeng H.; Sun H. Solution-Processed Low Threshold Vertical Cavity Surface Emitting Lasers from All-Inorganic Perovskite Nanocrystals. Adv. Funct. Mater. 2017, 27, 1605088 10.1002/ADFM.201605088.

[ref10] Chen S.; Zhang C.; Lee J.; Han J.; Nurmikko A.; Chen S.; Lee J.; Zhang C.; Han J.; Nurmikko A. High-Q, Low-Threshold Monolithic Perovskite Thin-Film Vertical-Cavity Lasers. Adv. Mater. 2017, 29, 1604781 10.1002/ADMA.201604781. 28211117

[ref11] Wang L.; Meng L.; Chen L.; Huang S.; Wu X.; Dai G.; Deng L.; Han J.; Zou B.; Zhang C.; Zhong H. Ultralow-Threshold and Color-Tunable Continuous-Wave Lasing at Room-Temperature from in Situ Fabricated Perovskite Quantum Dots. J. Phys. Chem. Lett. 2019, 10, 3248–3253. 10.1021/ACS.JPCLETT.9B00658/SUPPL_FILE/JZ9B00658_LIVESLIDES.MP4. 31084011

[ref12] Jia Y.; Kerner R. A.; Grede A. J.; Rand B. P.; Giebink N. C. Continuous-Wave Lasing in an Organic–Inorganic Lead Halide Perovskite Semiconductor. Nat. Photonics 2017, 11, 784–788. 10.1038/s41566-017-0047-6.

[ref13] Roh K.; Zhao L.; Rand B. P. Tuning Laser Threshold within the Large Optical Gain Bandwidth of Halide Perovskite Thin Films. ACS Photonics 2021, 8, 2548–2554. 10.1021/ACSPHOTONICS.1C00910/SUPPL_FILE/PH1C00910_SI_001.PDF.

[ref14] Pourdavoud N.; Haeger T.; Mayer A.; Cegielski P. J.; Giesecke A. L.; Heiderhoff R.; Olthof S.; Zaefferer S.; Shutsko I.; Henkel A.; Becker-Koch D.; Stein M.; Cehovski M.; Charfi O.; Johannes H. H.; Rogalla D.; Lemme M. C.; Koch M.; Vaynzof Y.; Meerholz K.; Kowalsky W.; Scheer H. C.; Görrn P.; Riedl T.; Pourdavoud N.; Haeger T.; Heiderhoff R.; Riedl T.; Mayer A.; Shutsko I.; Henkel A.; Scheer H. H.-C. H.; Görrn P.; Cegielski P. J.; Giesecke A. L.; Lemme M. C.; Olthof S.; Meerholz K.; Zaefferer S.; Becker-Koch D.; Vaynzof Y.; Stein M.; Koch M.; Cehovski M.; Charfi O.; Johannes H.-H. H. H.-H.; Kowalsky W.; Rogalla D. Room-Temperature Stimulated Emission and Lasing in Recrystallized Cesium Lead Bromide Perovskite Thin Films. Adv. Mater. 2019, 31, 1903717 10.1002/ADMA.201903717. 31402527

[ref15] Price M. B.; Lewellen K.; Hardy J.; Lockwood S. M.; Zemke-Smith C.; Wagner I.; Gao M.; Grand J.; Chen K.; Hodgkiss J. M.; Le Ru E.; Davis N. J. L. K. Whispering-Gallery Mode Lasing in Perovskite Nanocrystals Chemically Bound to Silicon Dioxide Microspheres. J. Phys. Chem. Lett. 2020, 11, 7009–7014. 10.1021/ACS.JPCLETT.0C02003/SUPPL_FILE/JZ0C02003_SI_001.PDF. 32786818

[ref16] Zhang H.; Liao Q.; Wu Y.; Zhang Z.; Gao Q.; Liu P.; Li M.; Yao J.; Fu H.; Zhang H.; Wu Y.; Yao J.; Fu H.; Liao Q.; Zhang Z.; Gao Q.; Liu P.; Li M. 2D Ruddlesden–Popper Perovskites Microring Laser Array. Adv. Mater. 2018, 30, 1706186 10.1002/ADMA.201706186. 29516558

[ref17] Tian X.; Wang L.; Li W.; Lin Q.; Cao Q. Whispering Gallery Mode Lasing from Perovskite Polygonal Microcavities via Femtosecond Laser Direct Writing. ACS Appl. Mater. Interfaces 2021, 13, 16952–16958. 10.1021/ACSAMI.0C21824/SUPPL_FILE/AM0C21824_SI_001.PDF. 33792289

[ref18] Qin C.; Sandanayaka A. S. D.; Zhao C.; Matsushima T.; Zhang D.; Fujihara T.; Adachi C. Stable Room-Temperature Continuous-Wave Lasing in Quasi-2D Perovskite Films. Nat 2020, 585, 53–57. 10.1038/s41586-020-2621-1. 32879501

[ref19] Athanasiou M.; Papagiorgis P.; Manoli A.; Bernasconi C.; Bodnarchuk M. I.; Kovalenko M. V.; Itskos G. Efficient Amplified Spontaneous Emission from Solution-Processed CsPbBr3Nanocrystal Microcavities under Continuous Wave Excitation. ACS Photonics 2021, 8, 2120–2129. 10.1021/ACSPHOTONICS.1C00565/SUPPL_FILE/PH1C00565_SI_001.PDF.

[ref20] Brenner P.; Bar-On O.; Jakoby M.; Allegro I.; Richards B. S.; Paetzold U. W.; Howard I. A.; Scheuer J.; Lemmer U. Continuous Wave Amplified Spontaneous Emission in Phase-Stable Lead Halide Perovskites. Nat. Commun. 2019, 10, 1–7. 10.1038/s41467-019-08929-0. 30816111 PMC6395683

[ref21] Evans T. J. S.; Schlaus A.; Fu Y.; Zhong X.; Atallah T. L.; Spencer M. S.; Brus L. E.; Jin S.; Zhu X. Y.; Evans T. J. S.; Schlaus A.; Zhong X.; Atallah T. L.; Spencer M. S.; Brus L. E.; Fu Y.; Jin S. Continuous-Wave Lasing in Cesium Lead Bromide Perovskite Nanowires. Adv. Opt. Mater. 2018, 6, 1700982 10.1002/ADOM.201700982.

[ref22] Jiang L.; Liu R.; Su R.; Yu Y.; Xu H.; Wei Y.; Zhou Z.-K.; Wang X. Continuous Wave Pumped Single-Mode Nanolasers in Inorganic Perovskites with Robust Stability and High Quantum Yield. Nanoscale 2018, 10, 13565–13571. 10.1039/C8NR03830A. 29974911

[ref23] Tian C.; Tong guo T. g.; Zhao S.; Zhai W.; Ge C.; Ran G. Low-Threshold Room-Temperature Continuous-Wave Optical Lasing of Single-Crystalline Perovskite in a Distributed Reflector Microcavity. RSC Adv. 2019, 9, 35984–35989. 10.1039/C9RA07442B. 35540621 PMC9074939

[ref24] Hsieh Y.-H.; Hsu B.-W.; Peng K.-N.; Lee K.-W.; Chu C. W.; Chang S.-W.; Lin H.-W.; Yen T.-J.; Lu Y.-J. Perovskite Quantum Dot Lasing in a Gap-Plasmon Nanocavity with Ultralow Threshold. ACS Nano 2020, 14, 11670–11676. 10.1021/ACSNANO.0C04224/SUPPL_FILE/NN0C04224_SI_001.PDF. 32701270

[ref25] Georgiou K.; Athanasiou M.; Jayaprakash R.; Itskos G.; Lidzey D. G.; Othonos A. Strong Light-Matter Coupling in Free-Standing Organic Membranes. J. Phys. Chem. C 2022. 10.1063/5.0178144 38112504

[ref26] Manoli A.; Papagiorgis P.; Sergides M.; Bernasconi C.; Athanasiou M.; Pozov S.; Choulis S. A.; Bodnarchuk M. I.; Kovalenko M. V.; Othonos A.; Itskos G. Surface Functionalization of CsPbBr3Νanocrystals for Photonic Applications. ACS Appl. Nano Mater. 2021, 4, 5084–5097. 10.1021/ACSANM.1C00558/SUPPL_FILE/AN1C00558_SI_001.PDF.

[ref27] Calzado E. M.; Villalvilla J. M.; Boj P. G.; Quintana J. A.; Díaz-García M. A. Tuneability of Amplified Spontaneous Emission through Control of the Thickness in Organic-Based Waveguides. J. Appl. Phys. 2005, 97, 093103 10.1063/1.1886891.

[ref28] Reufer M.; Feldmann J.; Rudati P.; Ruhl A.; Müller D.; Meerholz K.; Karnutsch C.; Gerken M.; Lemmer U. Amplified Spontaneous Emission in an Organic Semiconductor Multilayer Waveguide Structure Including a Highly Conductive Transparent Electrode. Appl. Phys. Lett. 2005, 86, 221102 10.1063/1.1938001.

[ref29] Navarro-Arenas J.; Suárez I.; Chirvony V. S.; Gualdrón-Reyes A. F.; Mora-Seró I.; Martínez-Pastor J. Single-Exciton Amplified Spontaneous Emission in Thin Films of CsPbX3 (X = Br, I) Perovskite Nanocrystals. J. Phys. Chem. Lett. 2019, 10, 6389–6398. 10.1021/ACS.JPCLETT.9B02369/SUPPL_FILE/JZ9B02369_SI_001.PDF. 31545904

[ref30] Yakunin S.; Protesescu L.; Krieg F.; Bodnarchuk M. I.; Nedelcu G.; Humer M.; De Luca G.; Fiebig M.; Heiss W.; Kovalenko M. V. Low-Threshold Amplified Spontaneous Emission and Lasing from Colloidal Nanocrystals of Caesium Lead Halide Perovskites. Nat. Commun. 2015, 6, 8056 10.1038/ncomms9056. 26290056 PMC4560790

[ref31] Grim J. Q.; Christodoulou S.; Di Stasio F.; Krahne R.; Cingolani R.; Manna L.; Moreels I. Continuous-Wave Biexciton Lasing at Room Temperature Using Solution-Processed Quantum Wells. Nat. Nanotechnol. 2014, 9, 891–895. 10.1038/nnano.2014.213. 25282045

[ref32] Anni M.; Cretí A.; De Giorgi M. L. D.; Lomascolo M. Local Morphology Effects on the Photoluminescence Properties of Thin Cspbbr3 Nanocrystal Films. Nanomaterials 2021, 11, 1470 10.3390/nano11061470. 34206075 PMC8227478

[ref33] Yuan X.; Hou X.; Li J.; Qu C.; Zhang W.; Zhao J.; Li H. Thermal Degradation of Luminescence in Inorganic Perovskite CsPbBr 3 Nanocrystals. Phys. Chem. Chem. Phys. 2017, 19, 8934–8940. 10.1039/C6CP08824D. 28300235

[ref34] Antlauf M.; Boulanger N.; Berglund L.; Oksman K.; Andersson O. Thermal Conductivity of Cellulose Fibers in Different Size Scales and Densities. Biomacromolecules 2021, 22, 3800–3809. 10.1021/ACS.BIOMAC.1C00643. 34510907 PMC8441976

[ref35] Asheghi M.; Touzelbaev M. N.; Goodson K. E.; Leung Y. K.; Wong S. S. Temperature-Dependent Thermal Conductivity of Single-Crystal Silicon Layers in SOI Substrates. J. Heat Transfer 1998, 120, 30–36. 10.1115/1.2830059.

[ref36] Mannino G.; Deretzis I.; Smecca E.; La Magna A.; Alberti A.; Ceratti D.; Cahen D. Temperature-Dependent Optical Band Gap in CsPbBr3, MAPbBr3, and FAPbBr3 Single Crystals. J. Phys. Chem. Lett. 2020, 11, 2490–2496. 10.1021/ACS.JPCLETT.0C00295/SUPPL_FILE/JZ0C00295_SI_001.PDF. 32148047 PMC7467746

[ref37] Tan M. J. H.; Chan Y.; H Tan M. J.; Chan Y. Pulsed Laser Photopatterning of Cesium Lead Halide Perovskite Structures as Robust Solution-Processed Optical Gain Media. Adv. Mater. Technol. 2020, 5, 2000104 10.1002/ADMT.202000104.

[ref38] Protesescu L.; Yakunin S.; Kumar S.; Bär J.; Bertolotti F.; Masciocchi N.; Guagliardi A.; Grotevent M.; Shorubalko I.; Bodnarchuk M. I.; Shih C.-J.; Kovalenko M. V. Dismantling the “Red Wall” of Colloidal Perovskites: Highly Luminescent Formamidinium and Formamidinium-Cesium Lead Iodide Nanocrystals. ACS Nano 2017, 11, 3119–3134. 10.1021/ACSNANO.7B00116/SUPPL_FILE/NN7B00116_SI_001.PDF. 28231432 PMC5800405

[ref39] Jin M.; Gao W.; Liang X.; Fang Y.; Yu S.; Wang T.; Xiang W. The Achievement of Red Upconversion Lasing for Highly Stable Perovskite Nanocrystal Glasses with the Assistance of Anion Modulation. Nano Res. 2021, 14, 2861–2866. 10.1007/S12274-021-3364-5.

[ref40] Papagiorgis P.; Manoli A.; Protesescu L.; Achilleos C.; Violaris M.; Nicolaides K.; Trypiniotis T.; Bodnarchuk M. I.; Kovalenko M. V.; Othonos A.; Itskos G. Efficient Optical Amplification in the Nanosecond Regime from Formamidinium Lead Iodide Nanocrystals. ACS Photonics 2018, 5, 907–917. 10.1021/ACSPHOTONICS.7B01159/SUPPL_FILE/PH7B01159_SI_001.PDF.

[ref41] Alvarado-Leaños A. L.; Cortecchia D.; Folpini G.; Kandada A. R. S.; Petrozza A. Optical Gain of Lead Halide Perovskites Measured via the Variable Stripe Length Method: What We Can Learn and How to Avoid Pitfalls. Adv. Opt. Mater. 2021, 9, 2001773 10.1002/ADOM.202001773/FORMAT/PDF.

[ref42] Qaid S. M. H.; Ghaithan H. M.; Al-Asbahi B. A.; Aldwayyan A. S. Achieving Optical Gain of the CsPbBr3Perovskite Quantum Dots and Influence of the Variable Stripe Length Method. ACS Omega 2021, 6, 5297–5309. 10.1021/ACSOMEGA.0C05414/SUPPL_FILE/AO0C05414_SI_001.PDF. 33681570 PMC7931209

[ref43] Shaklee K. L.; Leheny R. F. DIRECT DETERMINATION OF OPTICAL GAIN IN SEMICONDUCTOR CRYSTALS. Appl. Phys. Lett. 1971, 18, 475 10.1063/1.1653501.

[ref44] Georgiou K.; Jayaprakash R.; Lidzey D. G. Strong Coupling of Organic Dyes Located at the Surface of a Dielectric Slab Microcavity. J. Phys. Chem. Lett. 2020, 11, 9893–9900. 10.1021/ACS.JPCLETT.0C02751/SUPPL_FILE/JZ0C02751_SI_001.PDF. 33170714

[ref45] Yamashita K.; Takeuchi N.; Oe K.; Yanagi H. Simultaneous RGB Lasing from a Single-Chip Polymer Device. Opt. Lett. 2010, 35, 2451–2453. 10.1364/ol.35.002451. 20634860

[ref46] Athanasiou M.; Smith R. M.; Pugh J.; Gong Y.; Cryan M. J.; Wang T. Monolithically Multi-Color Lasing from an InGaN Microdisk on a Si Substrate. Sci. Rep. 2017, 7, 1–8. 10.1038/s41598-017-10712-4. 28855663 PMC5577231

[ref47] He H.; Cui Y.; Li H.; Shao K.; Chen B.; Qian G. Controllable Broadband Multicolour Single-Mode Polarized Laser in a Dye-Assembled Homoepitaxial MOF Microcrystal. Light: Sci. Appl. 2020, 9, 2577 10.1038/S41377-020-00376-7. PMC7424519 32821379

[ref48] Chellappan K. V.; Erden E.; Urey H. Laser-Based Displays: A Review. Appl. Opt. 2010, 49, F79–F98. 10.1364/ao.49.000f79. 20820205

[ref49] Fan F.; Turkdogan S.; Liu Z.; Shelhammer D.; Ning C. Z. A Monolithic White Laser. Nat. Nanotechnol. 2015, 10, 796–803. 10.1038/nnano.2015.149. 26214252

[ref50] Neumann A.; Wierer J. J.; Davis W.; Ohno Y.; Brueck S. R. J.; Tsao J. Y. Four-Color Laser White Illuminant Demonstrating High Color-Rendering Quality. Opt. Express 2011, 19, A982–A990. 10.1364/oe.19.00a982. 21747570

[ref51] Lin W.-Y.; Chen C.-Y.; Lu H.-H.; Chang C.-H.; Lin Y.-P.; Lin H.-C.; Wu H.-W. 410 m/500 Mbps WDM Visible Light Communication Systems. Opt. Express 2012, 20, 9919–9924. 10.1364/oe.20.009919. 22535084

[ref52] Lu Z.; Li Y.; Qiu W.; Rogach A. L.; Nagl S. Composite Films of CsPbBr3 Perovskite Nanocrystals in a Hydrophobic Fluoropolymer for Temperature Imaging in Digital Microfluidics. ACS Appl. Mater. Interfaces 2020, 12, 19805–19812. 10.1021/ACSAMI.0C02128/SUPPL_FILE/AM0C02128_SI_005.MP4. 32237718

[ref53] Athanasiou M.; Papagiorgis P.; Manoli A.; Bernasconi C.; Poyiatzis N.; Coulon P.-M.; Shields P.; Bodnarchuk M. I.; Kovalenko M. V.; Wang T.; Itskos G. InGaN Nanohole Arrays Coated by Lead Halide Perovskite Nanocrystals for Solid-State Lighting. ACS Appl. Nano Mater. 2020, 3, 2167–2175. 10.1021/ACSANM.9B02154.

[ref54] Furman S. A.; Tikhonravov A. V. Spectral Characteristics of Multi-Layer Coatings: Theory. In Basics of Optics of Multilayer Systems; Atlantica Séguier Frontières: Paris, 1992; pp 1–102.

