# Supplementary material for: Flexible, Free-Standing Polymer Membranes Sensitized by CsPbX3 Nanocrystals as Gain Media for Low Threshold, Multicolor Light Amplification
Source: ACS Photonics. 2022 Jun 24;9(7):2385–97. doi: 10.1021/acsphotonics.2c00426 (PMC9305998; doi:10.1021/acsphotonics.2c00426)
Supplement: Supplementary file 1 — ph2c00426_si_001.pdf [file ph2c00426_si_001.pdf]

Supplementary Information of

"Flexible, Free Standing Polymer Membranes Sensitized by $CsPbX_3$ Nanocrystals as Gain Media for Low Threshold, Multi-Color Light Amplification''

Modestos Athanasiou[†,], Andreas Manoli[†], Paris Papagiorgis[†], Kyriacos Georgiou[#], Yuliia Berezovska[‡], Andreas Othonos[#], Maryna I. Bodnarchuk[‡], Maksym V. Kovalenko[ǁ,‡] and Grigorios Itskos[†,*]*

[†]Experimental Condensed Matter Physics Laboratory, Department of Physics, University of Cyprus, Nicosia 1678, Cyprus

[ǁ]Institute of Inorganic Chemistry, Department of Chemistry and Applied Biosciences, ETH Zürich, CH-8093 Zürich, Switzerland

[‡]Laboratory for Thin Films and Photovoltaics, Empa – Swiss Federal Laboratories for Materials Science and Technology, Überlandstrasse 129, CH-8600 Dübendorf, Switzerland

[#]Department of Physics, Laboratory of Ultrafast Science, University of Cyprus, Nicosia 1678, Cyprus.

*itskos@ucy.ac.cy , *athanasiou.s.modestos@ucy.ac.cy

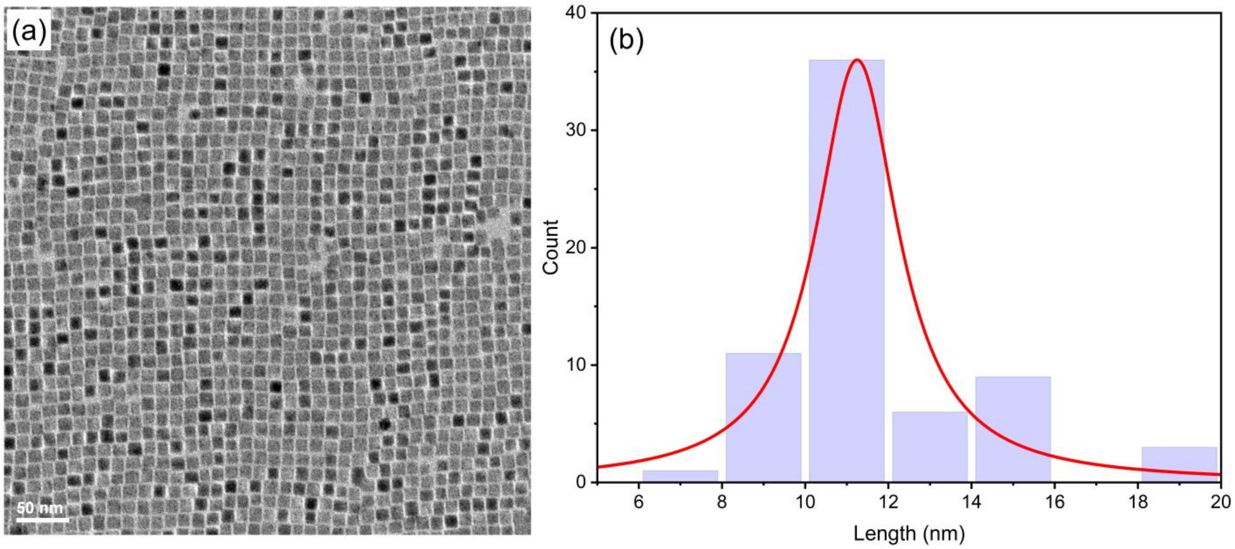

Figure S1. TEM images of (a) $CsPbBr_3$ NCs (b) size distribution histogram

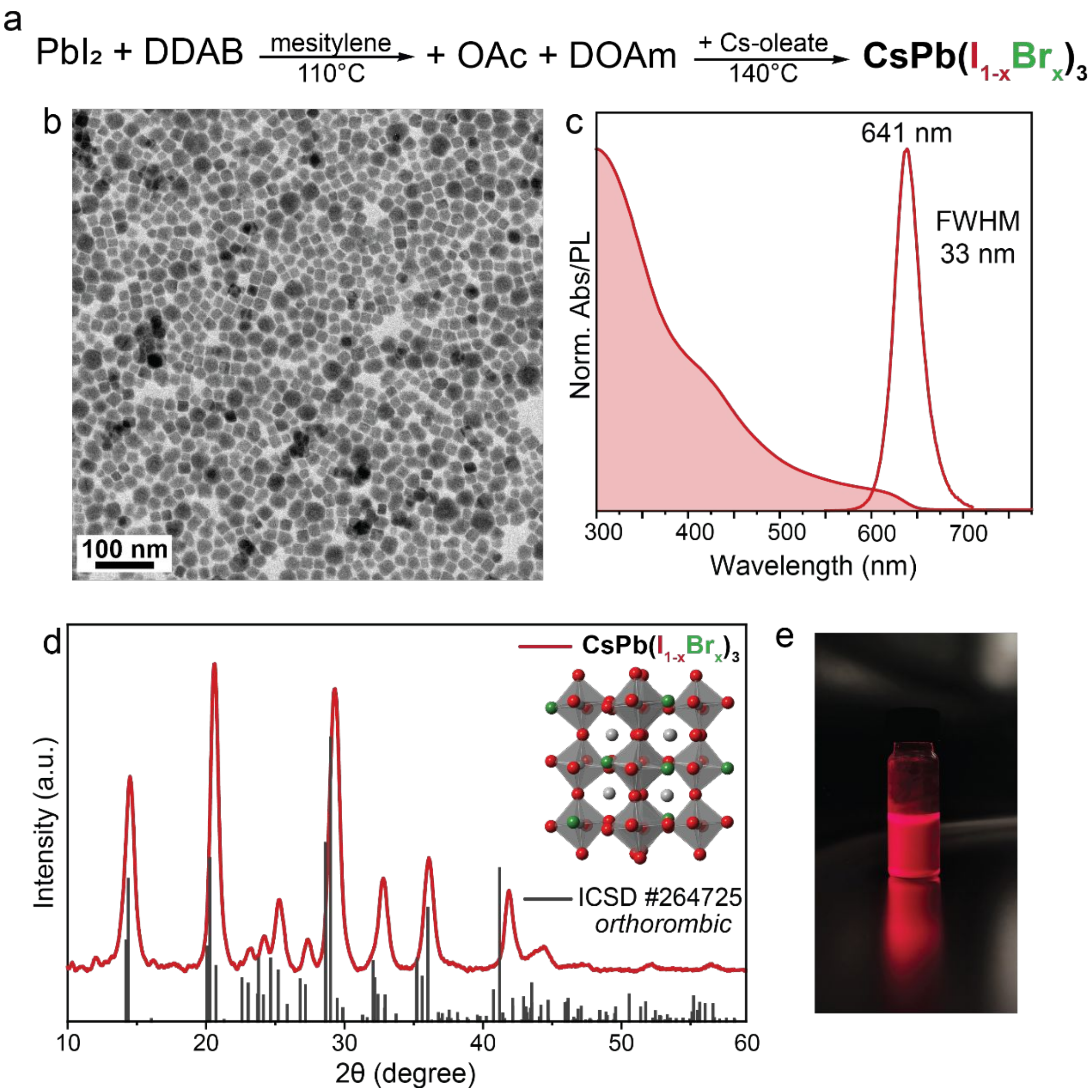

Figure S2. (a) Schematic illustration of the synthetic procedure, (b) TEM images and (c) representative room-temperature absorption and PL (normalized on excitonic peak) spectra of $CsPb(Br,I)_3$ NCs, (d) X-ray diffraction pattern of $CsPb(Br,I)_3$ NCs, and (d) the photograph of the colloidal solutions of $CsPb(Br,I)_3$ NCs under UV-light excitation, λ=365 nm.

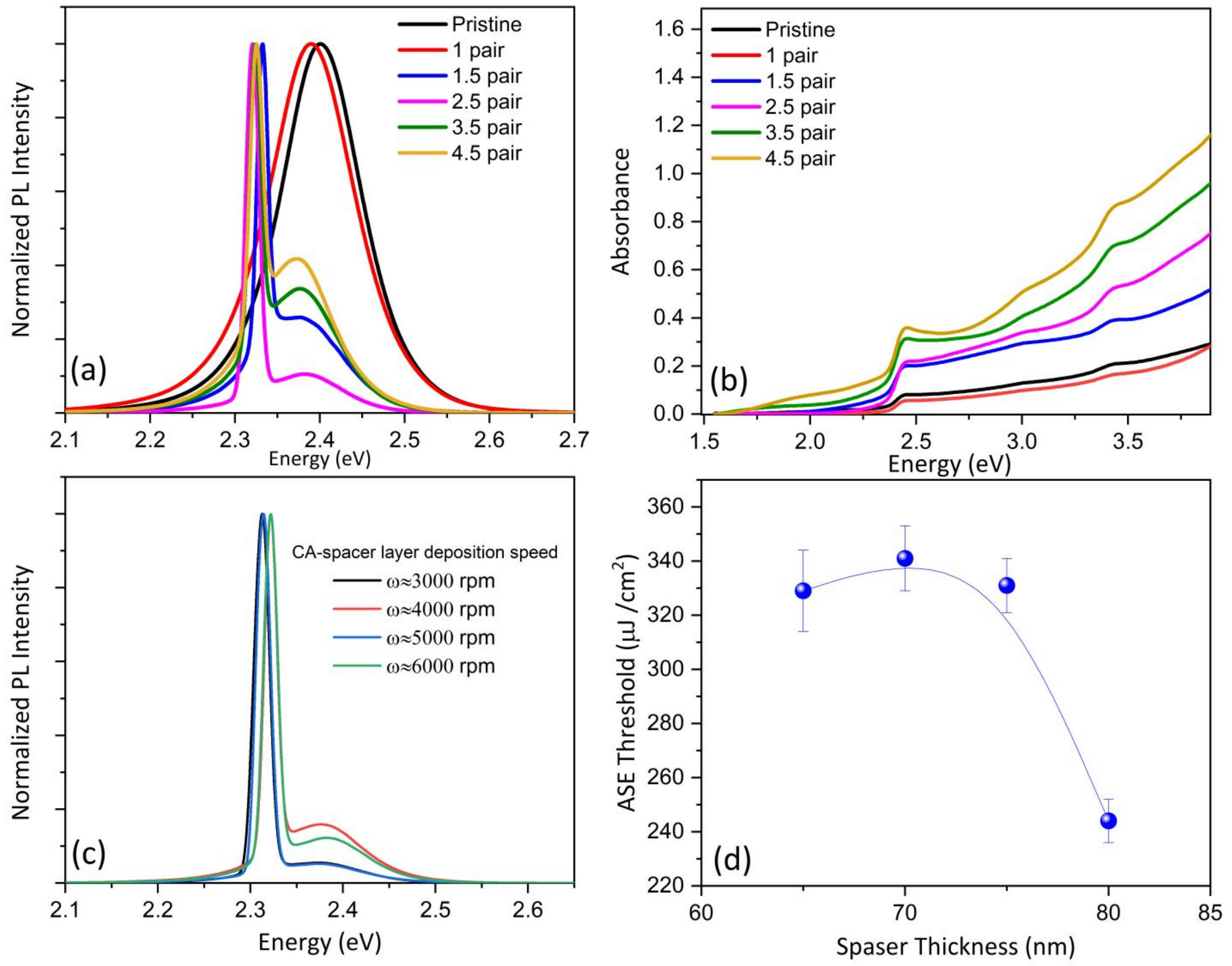

Figure S3. (a) Normalized emission spectra of $CsPbBr_3$ NC/CA multilayers deposited on quartz substrate. The NCs and CA concentration and deposition speed was kept the same during the experiments. Reference sample denotes a single layer deposition of CsPbBr3 NCs. (b) Absorbance as a function of number of NC/CA layers. (c) Normalized ASE spectra as a function of the polymer spacer thickness, while maintaining constant the deposition speed (3000 rpm) for $CsPbBr_3$ NCs and number of multilayers (2.5). (d) ASE threshold vs. the deposition speed of CA polymer. More detailed parametric study with respect of to the precise control of CA deposition via spin-casting method, can be found in our recently published work.[1]

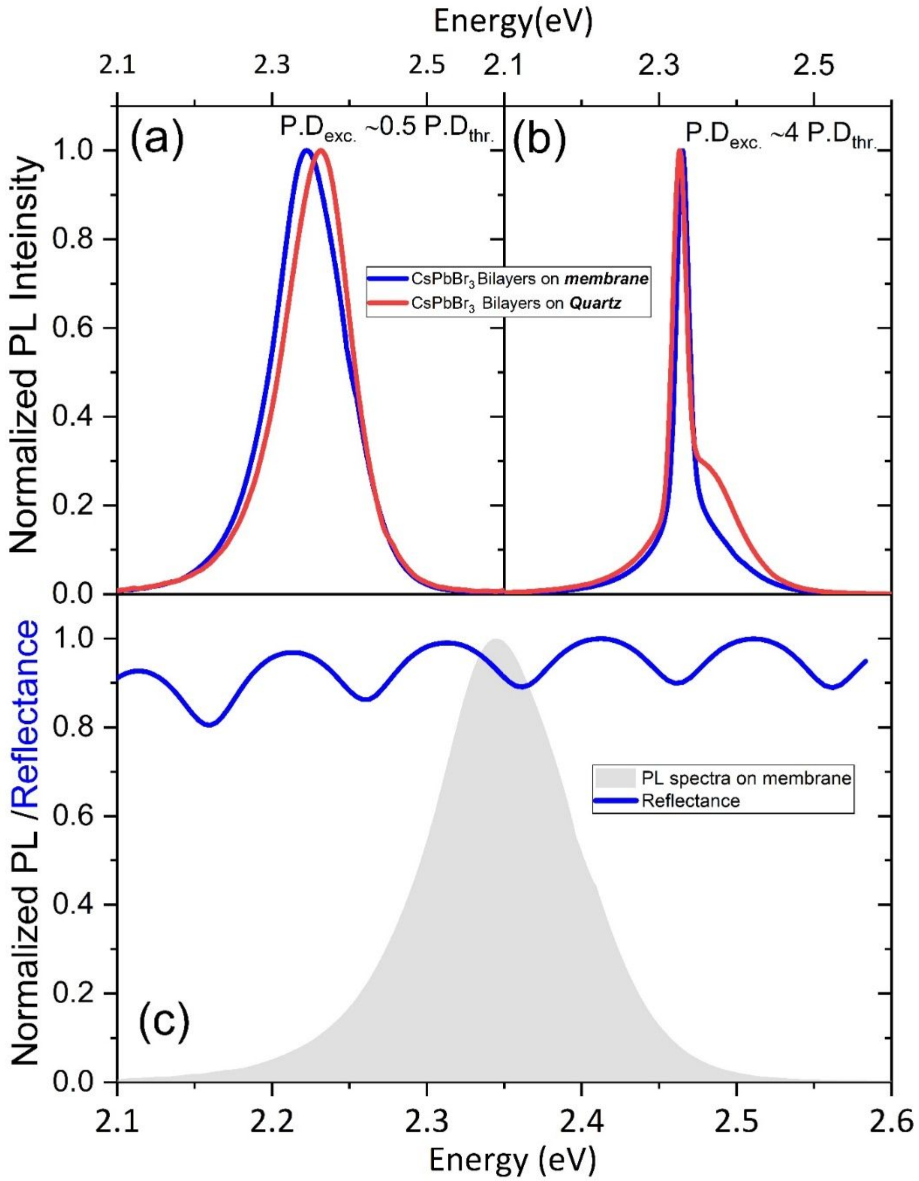

Figure S4. Normalized PL spectra of $CsPbBr_3$ NC/CA multilayers deposited on quartz and free-standing membrane (a) at ~0.5x the ASE threshold and (b) ~4x the ASE threshold. (c) Experimental PL data and TMM simulated reflectance spectra of the free-standing membrane.

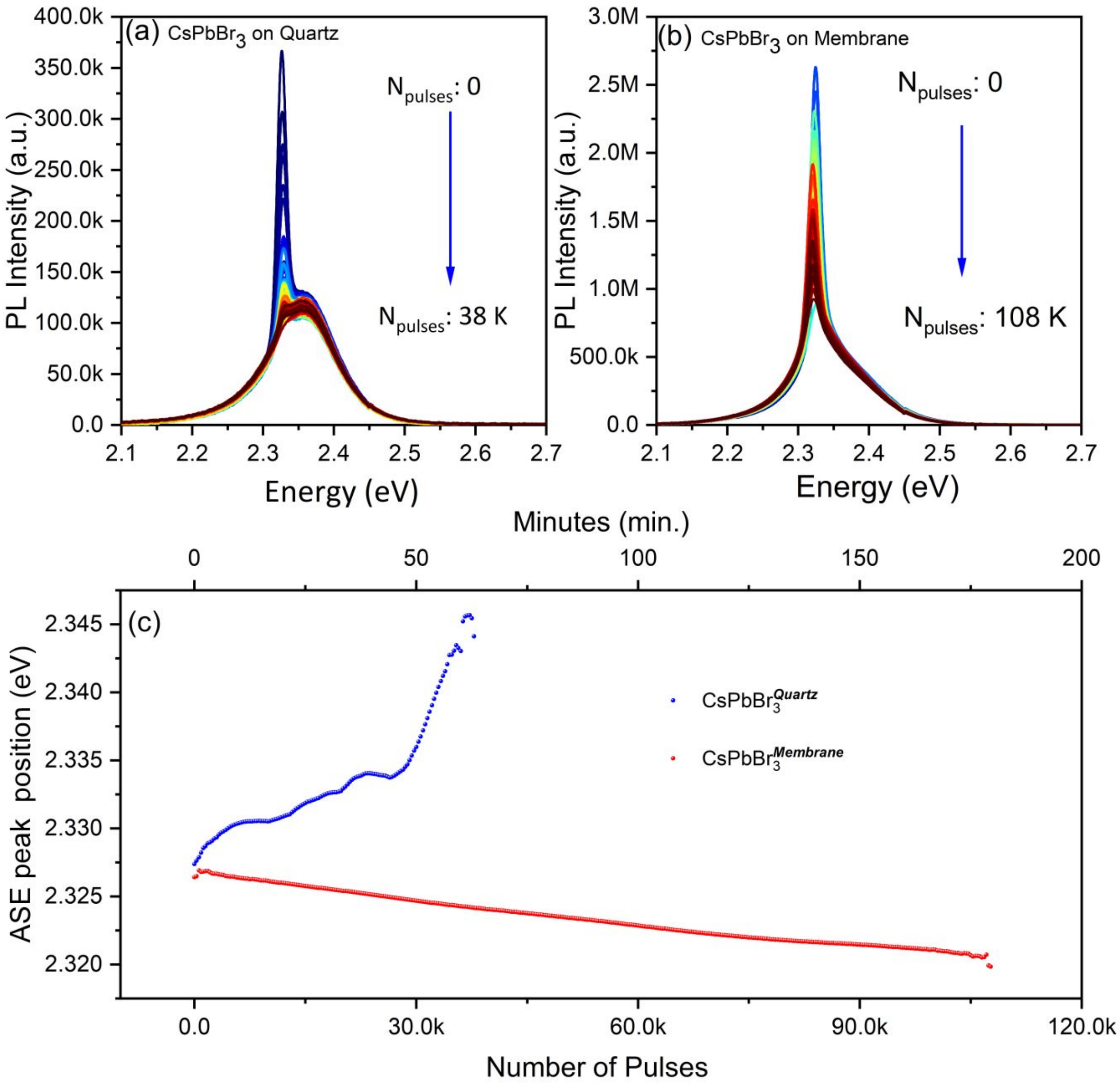

Figure S5. Evolution of ASE spectra of $CsPbBr_3$ NC/CA multilayers, (a) deposited on quartz and (b) on flexible membranes, as a function of the number of pulses at an excitation of 2 times the ASE threshold ($\times 2P_{th}$). The ASE peak position versus the number of pulses at the same excitation fluence.

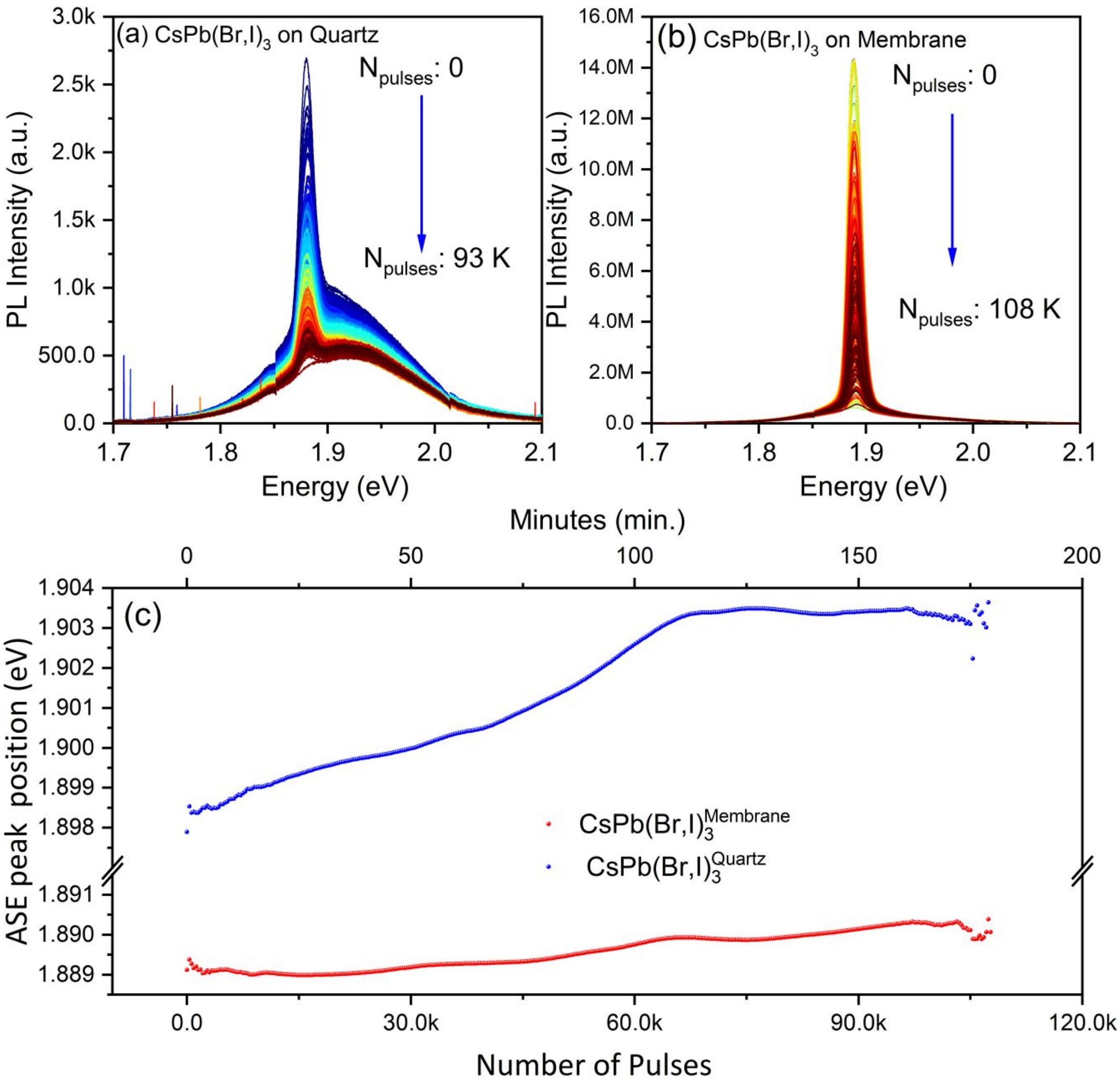

Figure S6. Evolution of ASE spectra of $CsPb(Br,I)_3$ NC/CA multilayers, (a) deposited on quartz and (b) on flexible membranes, as a function of the number of pulses at an excitation of 2 times the ASE threshold ($\times 2P_{th}$). The ASE peak position versus the number of pulses at the same excitation fluence.

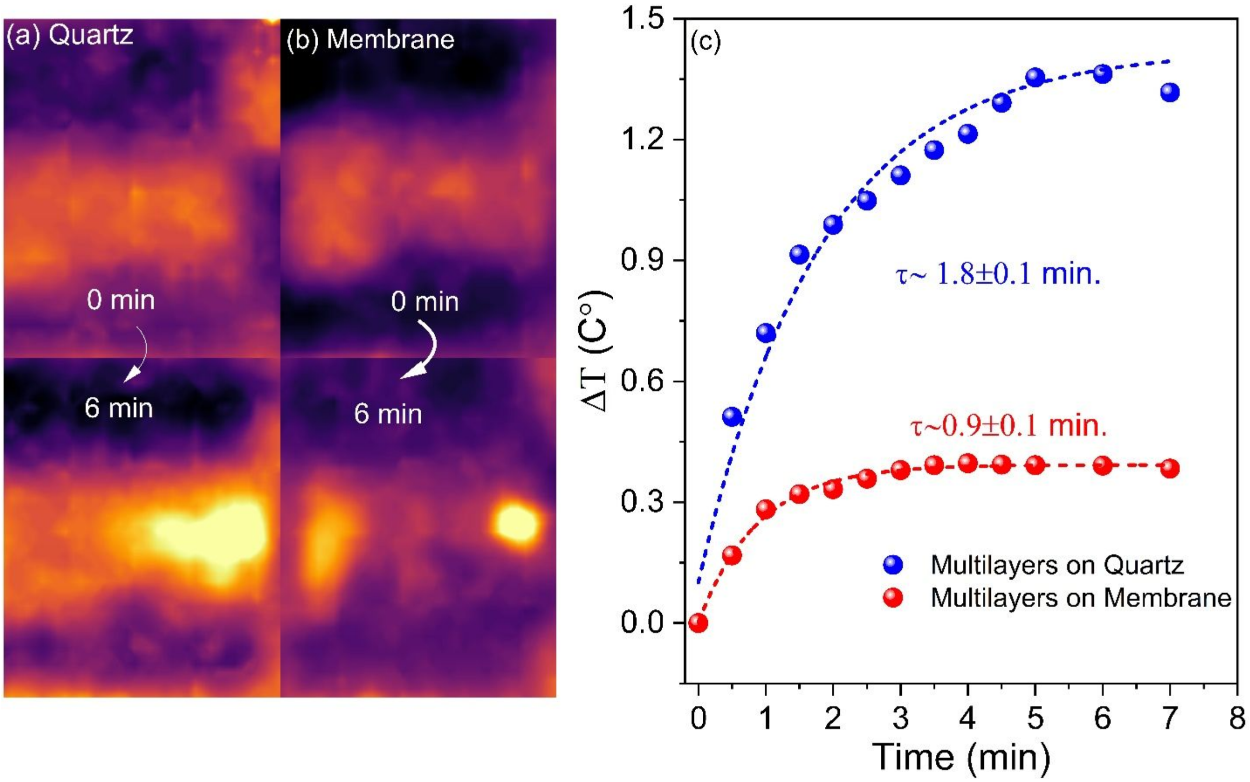

Figure S7. Thermal images of the samples under ASE operation for 6 minutes for (a) quartz and (b) flexible membranes. (c) Sample temperature rise (ΔT) for multilayers deposited on quartz and membrane, as a function of time. A single exponential fitting was used to extract the rise time (τ) of the temperature, with samples deposited on quartz showing a slower buildup of the temperature but with an overall temperature rise being ~3.5 times higher compared to the membranes.

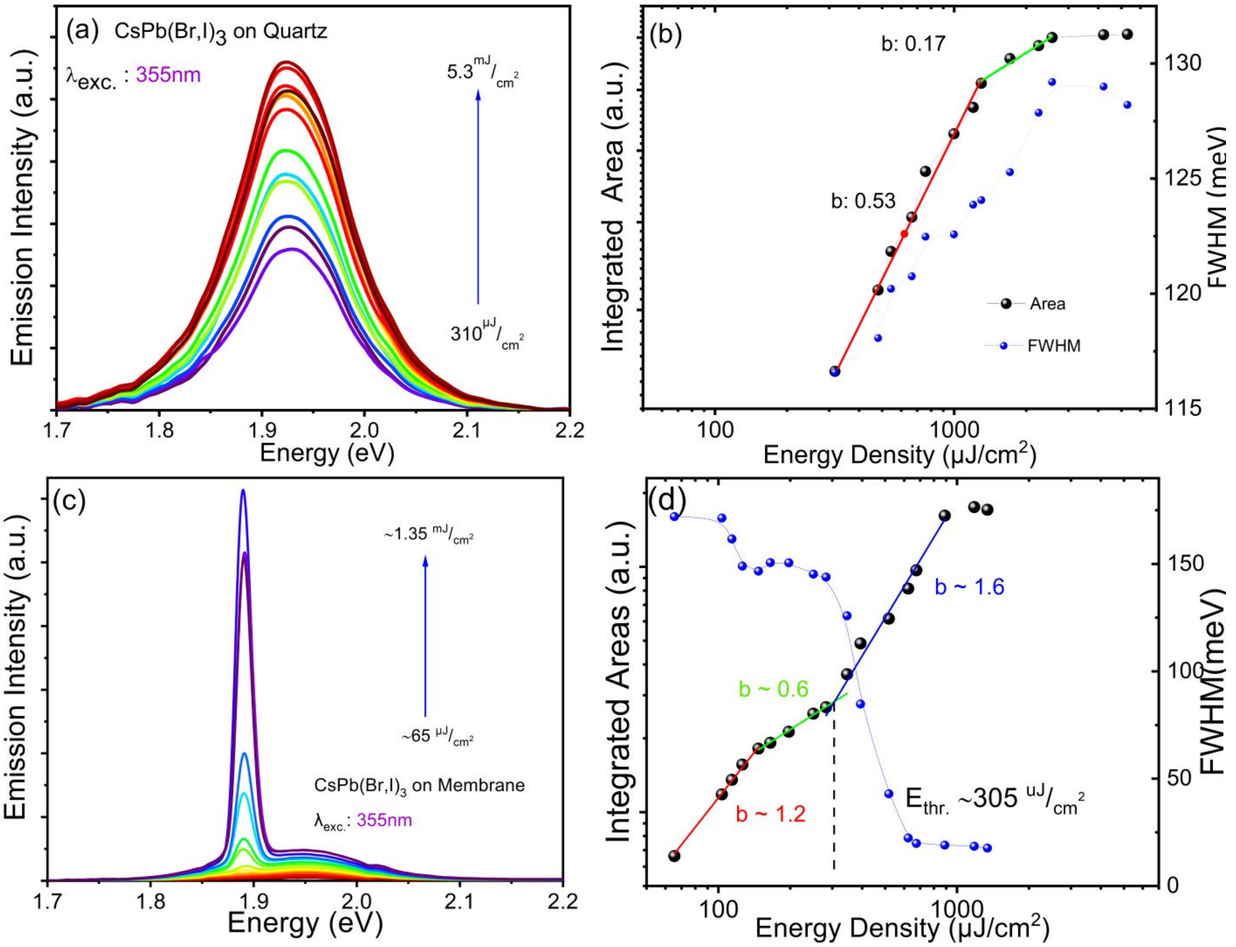

Figure S8. (a) PL spectra of $CsPb(Br,I)_3$ NCs and (b) Integrated area and FWHM of multilayers deposited on quartz as a function of excitation density under 355 nm nanosecond pulses. (c) Evolution of ASE spectra and (d) Integrated area and linewidth as a function of excitation density for multilayers deposited on flexible substrates.

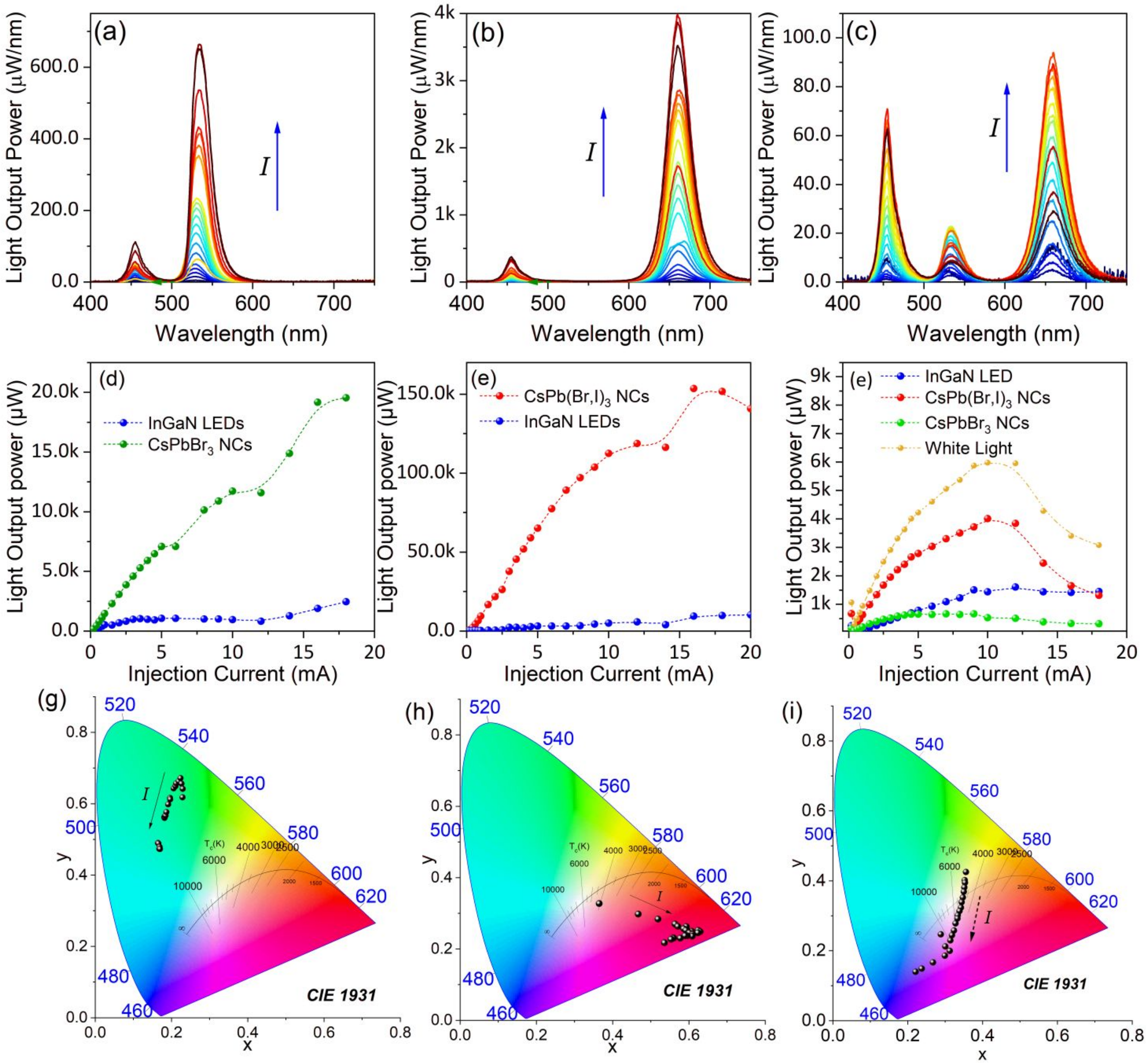

Figure S9. Electroluminescence spectra of a blue-emitting InGaN LED as function of current when coated with overlayers of: (a) $CsPbBr_3$ NCs (b) $CsPb(Br,I)_3$ NCs and (c) combination of both NCs. Integrated areas as a function of injection current when coating the LED with: (d) $CsPbBr_3$ NCs (e) $CsPb(Br,I)_3$ NCs and (f) combination of both NCs. CIE contour maps as a function of the current for LED with: (g) $CsPbBr_3$ NCs (h) $CsPb(Br,I)_3$ NCs and (i) combination of both NCs.

References:

1. Athanasiou M, Papagiorgis P, Manoli A, et al. Efficient Amplified Spontaneous Emission from Solution-Processed CsPbBr3Nanocrystal Microcavities under Continuous Wave Excitation. *ACS Photonics*. 2021;8(7):2120-2129. doi:10.1021/ACSPHOTONICS.1C00565/SUPPL_FILE/PH1C00565_SI_001.PDF
